# Electrochemical CO_2_ Valorization Pathways and Processes toward C_2_ to C_6_ Products: Acetylene, Propylene, Butadiene, and Benzene

**DOI:** 10.1021/acsenergylett.5c00467

**Published:** 2025-04-29

**Authors:** Jorge Ferreira de Araújo, Jan Rossmeisl, Hanqing Yin, Xingli Wang, Alexander Bagger, Peter Strasser

**Affiliations:** † Department of Chemistry, Chemical Engineering Division, Technical University Berlin, Straße des 17. June 124, 10623 Berlin, Germany; ‡ Department of Chemistry, University of Copenhagen, 2100 Copenhagen, Denmark; § Department of Energy, Technical University of Denmark, 2800 Kgs., Lyngby, Denmark; ∥ Department of Physics, Technical University of Denmark, 2800 Kgs., Lyngby, Denmark

## Abstract

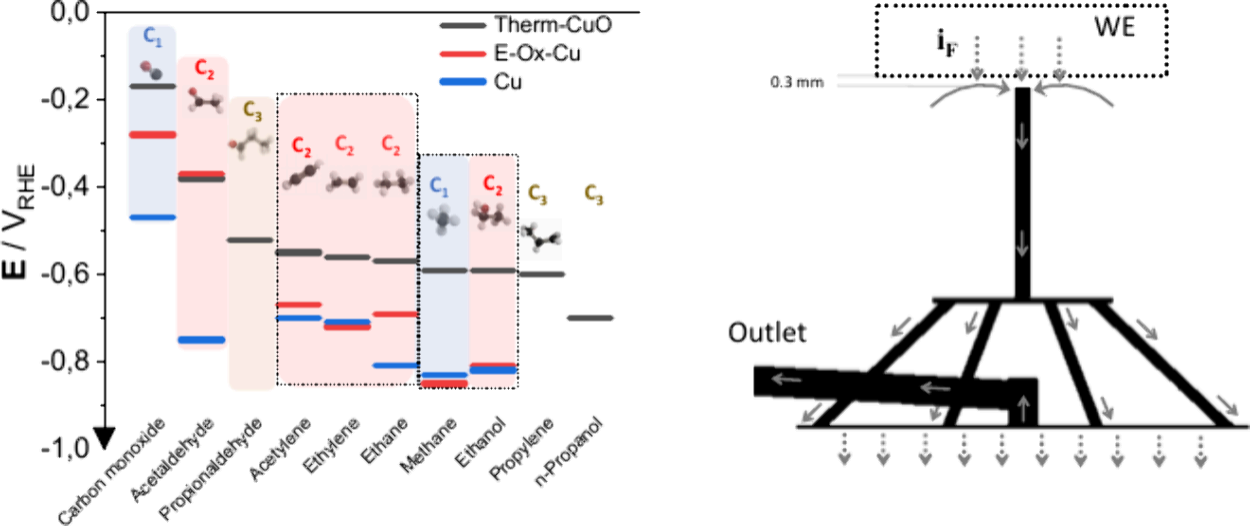

CO_2_ electrolysis on Cu catalysts at near-ambient conditions yields a range of important C_1_ to C_3_ products. Despite recent advances, our mechanistic understanding of the CO_2_ electrolysis reaction network has remained incomplete, with C_4_ products, and in particular long sought-after aromatic C_6_ product molecules, still being elusive. Here, we use a real-time capillary DEMS technique to determine the kinetic onset potentials of a wide set of C_1–3_ CO_2_ reduction products. Included in our study are rarely reported products, such as propionaldehyde, propylene, and, first, acetylene, C_2_H_2_. We then focus on the formation of acetylene, C_2_H_2_, and also investigate its alkyne electro-reduction, the C_2_H_2_ reduction reaction (C_2_H_2_RR). Acetylene electrodimerizes to the C_4_ compound 1,3-butadiene in a 2e^–^ reduction reaction. It also revealed a potential-dependent electroless Cu-catalyzed ambient-condition [2 + 2 + 2] cycloaddition reaction to C_6_ benzene. We discuss mechanisms and the significance of the potential-dependent valorizations of acetylene on Cu. We hypothesize a future process concept to valorize CO_2_ into sustainable C_6_ e-aromatics.

The low-temperature electrolysis of CO_2_ (the CO_2_ reduction reaction, CO2RR) using renewable electricity is an emerging Power-to-X technology that valorizes CO_2_ into molecular e-fuels and e-chemicals, while mitigating greenhouse gas emissions.^1−6^ CO_2_ electrolysis technologies target value-added carbon-neutral carbonaceous analogues of today’s fossil-derived platform chemicals. Yet to date, experimentally well-documented CO_2_ electrolysis products are largely limited to a set of C_1–3_ compounds.^5,6^ While small traces of linear C_4–6_ compounds have recently been reported,^7,8^ alkynes, nonaliphatic multiply unsaturated olefins, and cyclic aromatic C_4–6_ hydrocarbons—chemicals with major roles in organic process chemistry—have so far remained elusive. A low-temperature electrocatalytic CO_2_ reaction process to multiply unsaturated olefins (*e-dienes*) and *e-aromatics* constitutes an important advance in the CO_2_ electrolysis sciences, as it would open up new avenues for the electricity-based production of carbon-neutral aromatic chemicals as net-zero commodity chemicals for sustainable polymer or pharmaceutical industries.

Our current mechanistic understanding of CO_2_ electrolysis is largely based on experimental studies of quasi stationary CO2RR product formation rates. This is because most previous CO2RR studies have relied on time-delayed intermittent or offline analytical techniques, such as chromatography or nuclear magnetic resonance.^4−6,9^ Detection of the rates of change of product formation rates or short-lived reactive intermediates along the catalytic CO_2_ valorization cascade requires chemical analytics with higher time-resolution. For that purpose, differential electrochemical mass spectrometry (DEMS) was introduced as a powerful method to track the dynamic volatile product formation.^6,10−17^ A number of different differentially and nondifferentially pumped mass spectrometric cell, system, and electrode designs have been reported, each with distinct challenges and opportunities in terms of their analytical time delay and resolution, detectable mass range, and electrode geometry and environment.^6,10,13−27^

Previous work on the molecular reaction mechanism of CO_2_ electrolysis on Cu has established the dimerization of adsorbed *CO as the key elementary C–C coupling step toward C_2_ aliphatic and olefinic hydrocarbons and oxygenates.^5^ However, to date, there continues to exist a range of experimentally unvalidated mechanistic hypotheses, such as the generation of C_2_ alkyne-type surface intermediates (acetylene), the identification of selectivity limiting species for alcohol vs hydrocarbons, or the detailed experimental characterization of the emergence of competing aldehydes and olefins, such as propionaldehyde and propylene. A real-time experimental analysis of the point of first appearance of reactive intermediates along the CO_2_ valorization pathway during transient potential scans could help test mechanistic hypotheses, reveal new intermediates, exclude mechanistic alternatives, and unravel new reaction pathways and production processes. With an accurate knowledge of the electrode potentials where surface catalytic generation of product molecules sets in, the so-called *product onset potentials*,^26^ powerful electrode potential-product maps can ensue. To evaluate product onset potentials, real-time DEMS characterized by a continuous plug-flow product transfer and detection in the millisecond range is an experimental tool like no other.^6,13−15,17,21,26−30^ By contrast, standard analytical chromatographic or magnetic resonance techniques inherently fail to meet continuous plug-flow sampling detection conditions. Correlating onset potential-product maps to computational reaction networks holds the promise to uncover further molecular insights into catalytic CO_2_ reaction cascades.

This contribution explores the use of capillary-flow DEMS for real-time detection of poorly documented or rarely characterized reaction intermediates and products during electrochemical CO_2_ valorization on Cu. Our present DEMS approach offers innovations in electrolyte gas saturation, electrolyte pumping, capillary sampling, and gas/liquid extraction into the evacuated portion of the DEMS system. The system makes use of a rapid dual gas–liquid extractor technique that allows for simultaneous detection of a wide range of gases and liquids. The system combines benefits known from previous OLEMS approaches, such as electrode geometry flexibility and minimal mass transport limitations, with time resolution benefits of (dual) thin electrolyte-layer approaches. We utilize the DEMS system to explore short-lived intermediates during the reductive conversion of CO_2_. Our study provides evidence for the electrochemical valorization of CO_2_ into molecular acetylene, C_2_H_2_, a short-lived highly reactive intermediate. Acetylene showed a subsequent reductive electrode-dependent reductive dimerization to butadiene, C_4_H_6_, and an electroless/thermal conversion to molecular benzene, C_6_H_6_, via an unusual potential-dependent, yet electroless Cu-catalyzed cyclotrimerization, likely by means of a [2 + 2 + 2] cycloaddition. We have evidence that the potential dependence arises from catalytically inactive surface Cu oxides that reduce at cathodic polarization and give way to metallic Cu sites where trimerization can occur. Using the DEMS product onset potentials, we map out a large portion of the molecular CO_2_ valorization cascade and discuss its mechanistic implications.

## Real-time Capillary DEMS Analysis of CO_2_ Reduction Products

**Figure 1:**
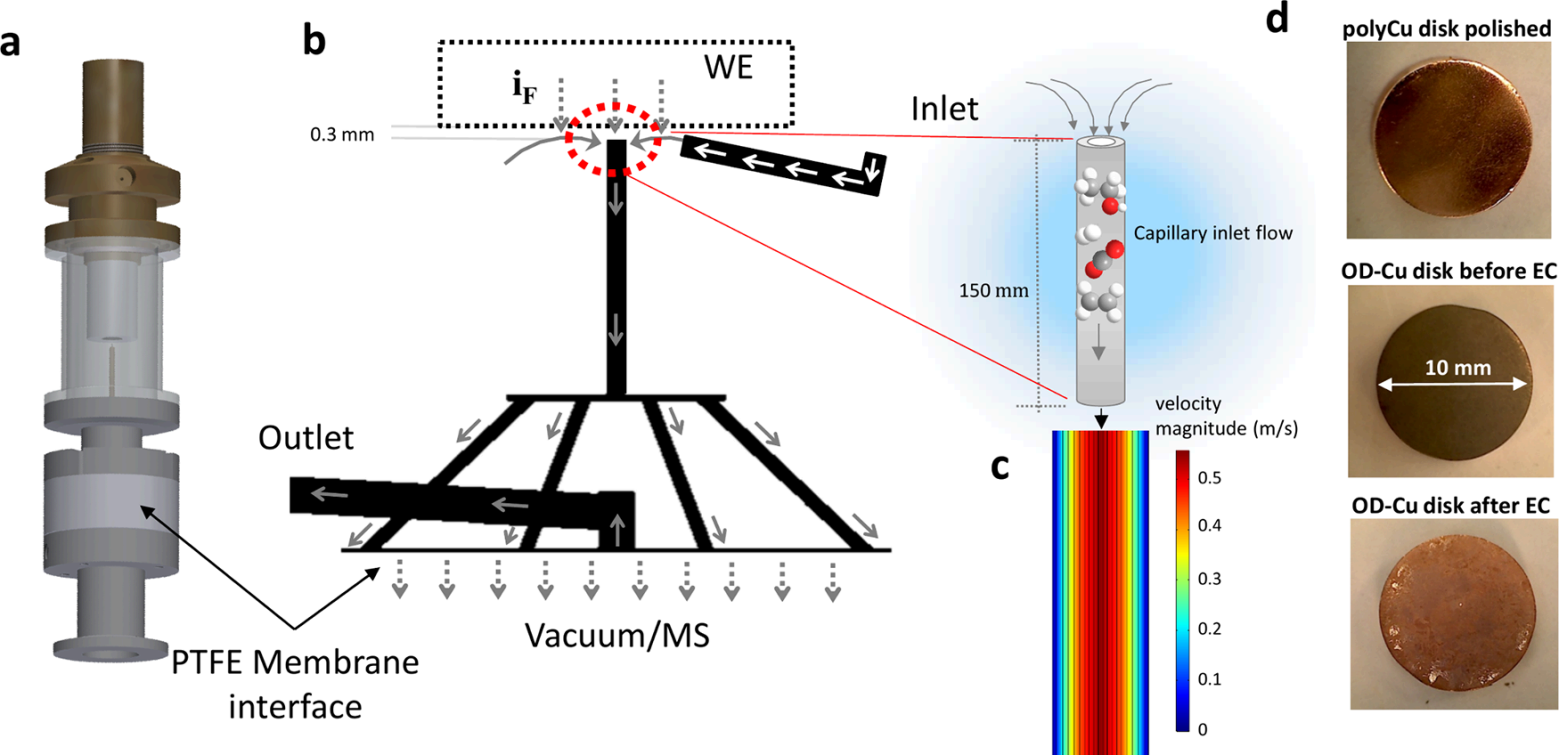
The electrochemical capillary-flow electrolyte-vacuum cell interface, a component of the differential electrochemical mass spectrometer (MS) system (see Figures S2–S5). (a) Schematic setup of the overall electrochemical cell body with embedded capillary inlet and liquid-vacuum extractor. (b) Enlarged scheme of liquid electrolyte flow into main flow cell compartment and over the working electrode (WE) surface during faradaic current flow – i_F_, sampling through the capillary positioned at distance of 0.3 mm from WE surface, collection of electrolyte near electrode surface and distribution over 4 capillaries to the PTFE membrane interface extractor to the evacuated region of the MS. Enlarged scheme shows tip of collection capillary. (c) Computational fluid dynamics (CFD) simulation of flow velocity distribution and streamlines. For calculation details, see the SI. (d) Polycrystalline copper-based disk electrodes (Ø 10 mm) employed in this study: (top) as polished before thermal or electrochemical treatment (referred to as “Cu”); (center) oxidized copper disk electrode after thermal oxidation at 500 °C for 10 min in 20% O_2_/80% Ar atmosphere (referred to as “Therm-CuO”); (bottom) reduced Therm-CuO disk electrode after electrochemical CO_2_ reduction in 0.1 M potassium bicarbonate solution.

Figure 1 illustrates the overall design of the flow-through capillary differential electrochemical mass spectrometry (DEMS) cell (Figure 1a), the detailed capillary electrolyte flow path characteristics (Figure 1b,c), and Cu-based catalytic electrodes employed in this study (Figure 1d and Figure S1). The cylindrical 10 mm diameter bulk Cu working electrode (WE) was mounted face-down and immersed into a large electrolyte volume in order to minimize mass transport limited operation regimes often encountered in thin-layer DEMS cell designs. Continuous electrolyte inflow from the right (white arrows) supplies fresh electrolyte to the catalytic active WE surface. A 150 μm diameter capillary orifice is located about 300 μm off the electrified catalytic solid–liquid interface and continuously samples electrolyte from the interface at a rate of 2–5 μL s^–1^. The capillary electrolyte flow passes various splitters before volatile reactant and product molecules cross the porous electrolyte–vacuum membrane interface into the differentially pumped mass spectrometer system (Figure S2). Figure 1c and Figure S3 present fluid dynamics simulations of the electrolyte flow velocity distribution inside and along the capillary. Driven by hydrostatic pressure differences between inside and outside the DEMS cell, electrolyte flow velocities reach and exceed 0.5 m s^–1^, which results in a millisecond convective transfer to the liquid–vacuum interface. Figure S4 provides more details on the capillary cell sampling and volatile species extraction system. This DEMS system offers a dual extraction system with separate extractors for liquid and gas molecules. Capillary cells and a vacuum system are embedded into the overall DEMS system schematically shown in Figure S5. All liquid flows are enabled by hydrostatic pressure differences avoiding syringe or peristaltic pumping, which would compromise a steady ion mass background current. Aside from the capillary sampling system, a novel dual inline saturation stack component (Figure S6) ensures a homogeneous, fast, and reliable gas saturation of the electrolyte. A Teflon membrane interfaces the flow-through gas and liquid compartments, thereby offering excellent control over the dissolved gas concentration. Overall, while quantifying molecular detection continues to require careful calibration and signal normalization using relative sensitivity factors (RSFs),^31−34^ our novel capillary DEMS system combines the high faradaic currents and the flexibility of OLEMS^35−37^ systems in terms of the geometry and chemical nature of the electrocatalyst to be investigated (i.e., single crystals, polycrystalline substrates, powder thin film catalysts etc.) with the time resolution of (dual) thin-layer DEMS^13,15,17^ designs. Unlike earlier OLEMS architecture, in the present capillary sample system the liquid–vacuum membrane interface is located far from the sample probe inlet and, thus, far from the catalytic interface; however, it is designed as a rapid flow-through system in order to achieve high time resolutions.

## Real-time DEMS Extraction of Onset Potentials of Key C_1_ and C_2_ Products

**Figure 2:**
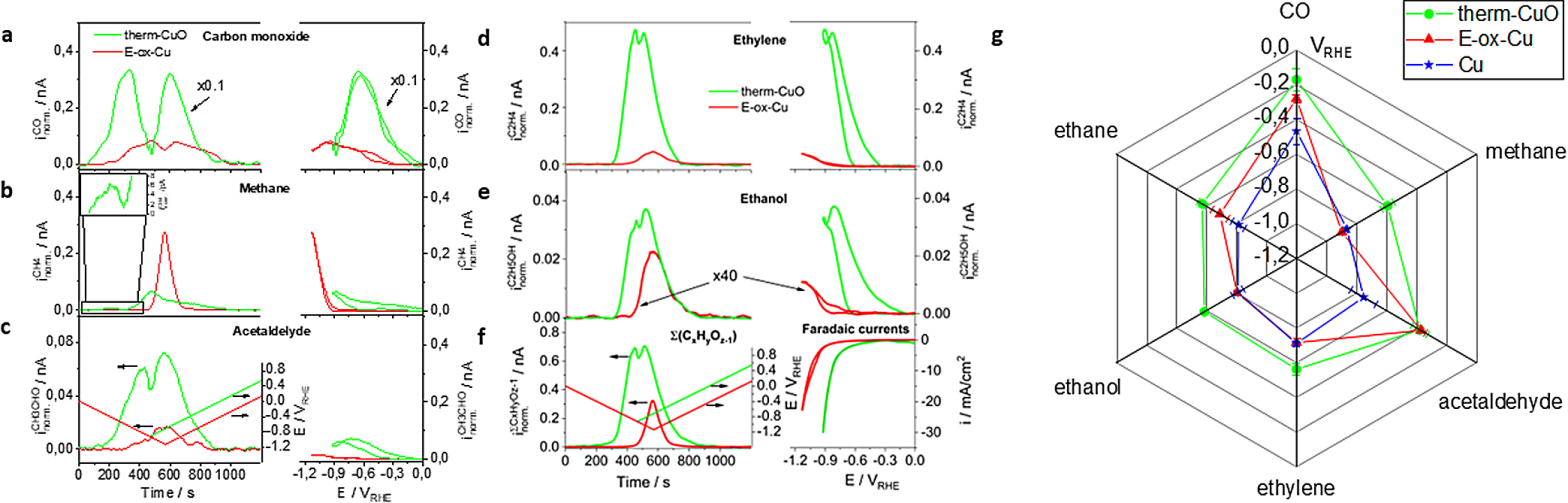
DEMS analysis and product onset potentials. (a–f) Mass spectrometric cyclic voltammograms (MSCV) of selected volatile gas and liquid products observed during the CO_2_ electroreduction reaction (CO2RR), plotted against time (left plots, left *y*-axis in panels a–f) and plotted against electrode potential (right plots, right *y*-axis in panels a–f) recorded on a thermally oxidized copper disk electrocatalyst (“therm-CuO”, green lines) and an electrochemically oxidized copper disk catalyst (“E-ox-Cu”, red lines) during one potential cycle (see panels c and f) into the catalytically active region of the CO2RR in 0.1 M KHCO_3_ electrolyte. Shown are MSCVs of the normalized mass currents (*i*_Norm_) of the 100% intensity fragment: (a) Carbon monoxide (CO) plotted using *m*/*z* = 28 fragment CO+ (100%); (b) Methane (CH_4_) at *m*/*z* = 16 (100%), plotted using *m*/*z* = 15 fragment CH_3_+ (85%), inset of b: transient early methane detection on therm-CuO; (c) Acetaldehyde plotted using *m*/*z* = 29 fragment CHO+ (100%), left plot, right *y*-axis: Applied triangular voltammetric potential cycles for the two catalysts; (d) Ethylene (C_2_H_4_) at *m*/*z* = 28 (100%), here plotted using*m*/*z* = 26 fragment C_2_H_2_+ (55%); (e) Ethanol (EtOH) plotted using *m*/*z* = 31 fragment CH_2_OH+ (100%). (f) left plot, left *y*-axis: Comparison of sum of all C_1+2_ product MSCVs of therm-CuO and E-ox-Cu; left plot, right *y*-axis: Applied triangular voltammetric potential cycle for the two catalysts. Right plot, right *y*-axis: Experimental faradaic current density against applied electrode potential (polarization curves). Other experimental parameters: scan rate 2 mVs^–1^, CO_2_-saturated electrolyte with *p*_CO_2__ = 100 kPa. (g) Radar plot of the experimental onset potential values (V_RHE_) of products extracted from the MSCVs in (a–f) of the therm-CuO catalyst (green circles), of the E-ox-Cu catalyst (red triangles), and a polished polycrystalline Cu reference catalyst (blue stars, see MSCVs in Figure S7). The onset potential was defined as the potential where ion mass currents reached 1% of peak value during the cathodic sweep.

Widely employed analytical techniques in CO_2_ reduction, such as nuclear magnetic resonance (NMR) or chromatographic methods (GC, HPLC) do not offer the required analytical time resolution in order to extract reliable onset electrode potentials of the CO_2_ reduction reaction products. To address this unmet need, the present capillary DEMS system was used to extract the onset electrode potentials of a set of previously well-documented as well as novel reaction products. For the present study we prioritized Cu-based surfaces over other catalytic surfaces, such as Ni or Co.^38,39^. This choice was based on the generally larger faradaic hydrocarbon efficiencies of Cu-based surfaces. The product onset potentials were evaluated for three distinct Cu-based catalytic surfaces prepared from Cu cylinders (Figure 1d and Figure S1): first, a polished, electroreduced metallic Cu surface (labeled “Cu”) featuring a metallic Cu(0) redox state; second, an electrochemically oxidized CuO_*x*_ surface (“E-ox-Cu”) revealing a dominant Cu(I) redox state; and third, a thermally air-oxidized CuO surface (labeled “therm-CuO”) characterized by a Cu(II) redox state (Supplementary Figure S1). To follow the evolution of CO_2_ reduction products, the most intense 100% mass-overcharge peaks were chosen, where possible (Supplementary Table 1). In the case of peak overlap, such as for *m*/*z* = 28 for ethylene, carbon monoxide, and the second most intense CO_2_ fragment, individual contributions were extracted by mass peak deconvolution (see Supplementary Information sections 1.1.5–1.1.8). Mass spectrometric cyclic voltammograms (MSCVs) of six major *C*_1/2_ reaction products (CO, CH_4_, CH_3_CHO, C_2_H_6_, C_2_H_4_, CH_3_OH) plotted in the time and potential domain for each of the three Cu-based catalytic surfaces are reported in Figure 2a–f and Supplementary Figure S7. A triangular potential scan was applied in order to track the onset of the products and evaluate their onset potentials. The lower turning potentials (see Figure 2c) were chosen such that the maximum cathodic current density (see Figure 2f) for each catalyst remained in the 20–30 mA cm^–2^ range. Due to the sharply distinct catalytic reactivity of the three surfaces, that lower turning potential ranges from −1 to −1.2 V_RHE_. Figure 2 and Supplementary Figure S7 demonstrate the dramatic catalytic activity and C_2_ product formation rate and yield increase, when moving from an initially metallic Cu, to a surface-oxidized CuO_*x*_, and to a thermally oxidized CuO surface. The excellent C_2_ production formation of initially thermally oxidized CuO has been well documented over the past decade:^5,40,41^ in situ reduction of the CuO surface to the metallic state results in oxide-derived (OD) Cu surfaces. Interestingly, the production of the C_1_ hydrocarbon, methane, remains strongly suppressed on the therm-CuO surface compared to the other two surfaces (Figure 2b), in line with previous hypotheses that low-coordinated OD-Cu sites are conducive for C–C coupling by CO dimerization. Supplementary Figure 8 directly contrasts all three catalytic surfaces and corroborates the C_2_ productivity of the oxide-derived therm-CuO catalyst. Supplementary Figures 9 and 10 detail the stability of the MSCV profiles over 20 consecutive scans for each product.

In the next step, we defined the onset potential for each CO_2_ reduction product as the electrode potential during the cathodic sweep, where the measured faradic current reached 1% of its peak current value. Figure 2g shows a radar plot of the experimental onset potentials of the six major C_1_ and C_2_ products for each of the three catalytic surfaces (therm-CuO in green, E-ox-Cu in red, and Cu in blue). Supplementary Table 2 provides the precise onset potential values. The outer edge denotes more positive onset electrode potentials and, hence, more energy efficient product generation. The therm-CuO onset potentials were found to be more positive (lower overpotentials) for essentially all six products, followed by the E-ox-Cu and the metallic Cu surface. Surprisingly, while acetaldehyde displayed sharply more negative onset potentials on metallic Cu, its production appears to be quite independent of the nature of the oxidative pretreatment. Ethanol and ethylene required the initial thermal oxidation of Cu to CuO in order to display onset potential benefits over metallic Cu. Supplementary Figure 11 details the initial onset potentials of six major products and tracks their temporal evolution over 12 consecutive potential cycles for the E-ox-Cu catalyst. The experiments revealed sustained similar, though slightly fluctuating, onset potentials for acetaldehyde and ethylene, which strongly suggests that acetaldehyde is not a molecular precursor for ethylene. Ethanol on the other hand showed a 200 mV negative onset potential shift vs acetaldehyde, pointing to a mechanistic acetaldehyde–ethanol reaction cascade. Methane generation displayed sustained similar onset potentials as ethanol, a fact that we had previously reported and linked to a hypothesis whether there exists surface C–C-coupling pathway between a methyl and a C(H)O fragment.^42^ As the availability of methyl surface species is limited by the protonation of *C(H)O at rather cathodic electrode potentials where methane is formed,^5^ such C–C coupling products would be expected to emerge in concomitance of methane. To date, this hypothesis awaits its confirmation or rejection.

## DEMS Detection of C_3_ Products: Propylene, Propionaldehyde, Propanol

**Figure 3:**
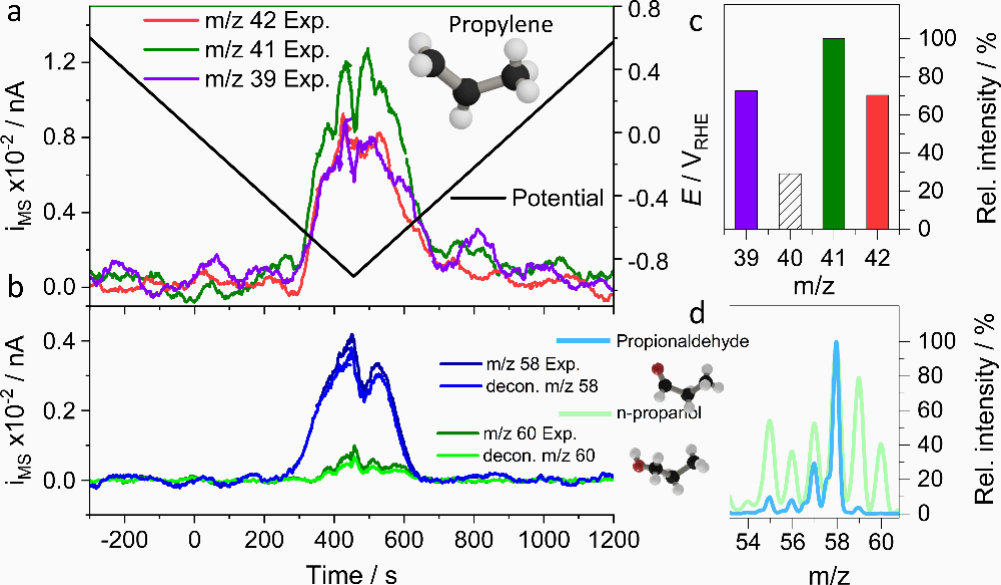
CO_2_ electroreduction to C_3_ compounds: Mass spectrometric cyclic voltammograms (MSCV) in the time domain for the identification of liquid and gaseous C_3_ hydrocarbons (propylene) and C_3_ oxygenate compounds (propionaldehyde, propanol) formed on the therm-CuO catalyst during a CO2RR voltammetric potential scan using a quantitative analysis of multiple ion mass spectrometric signals. (a) DEMS evidence and onset potentials of propylene using the ion mass intensities (left *y*-axis) of the characteristic fragments (cf. panel c) at *m*/*z* = 39 (purple), *m*/*z* = 41 (green), and *m*/*z* = 42 (red). Right *y*-axis: Applied electrode potential (black line); (b) DEMS evidence and onset potentials of C_3_ oxygenates 1-propanol and propionaldehyde by deconvolution of the experimental MSCV profiles at characteristic fragments (cf panel d) at *m*/*z* = 58 and 60 (dark blue and dark green lines). Deconvoluted propionaldehyde MSCV profiles at *m*/*z* = 58 (navy blue line) and 1-propanol MSCV profiles at *m*/*z* = 60 (light green line). (c) Selected characteristic mass fragments of pure propylene feeds on therm-CuO used in panel a; (d) Experimental reference mass fragmentation spectra of liquid cofeeds of propionaldehyde (blue) and 1-propanol (green). Experimental CO2RR conditions: CO_2_-saturated 0.1 M KHCO_3_ (*P*_CO_2__ = 100 kPa). A triangular voltammetric cycle was applied starting at +0.54 V_RHE_ to −0.9 V_RHE_ at 2 mVs^–1^ (see right *y*-axis in panel a).

The real-time capillary DEMS system proved its strong power during the analysis of C_3_ CO_2_ reduction products on the therm-CuO catalyst. Figure 3a,c details the experimental MSCVs in the time domain of the three characteristic *m*/*z* (39, 41, 42) peak patterns of the important, yet rarely discussed, chemical product propylene. The applied cyclic potential sweep is shown in black. While propylene has rarely been documented before, this is the first time that it was possible to accurately determine its onset potential (see Supplementary Table 2). Similarly, we determined the generation and onset potential of more common C_3_ compounds, such as propionaldehyde^25^ and n-propanol (Figure 3b,d). Their overlapping peak patterns required careful deconvolution of ion masses 58 and 60. Note that C_3_ products were only detectable on the oxide-derived therm-CuO catalyst.

## DEMS-Based Discovery of the Acetylene C_2_ Alkyne Intermediate

**Figure 4:**
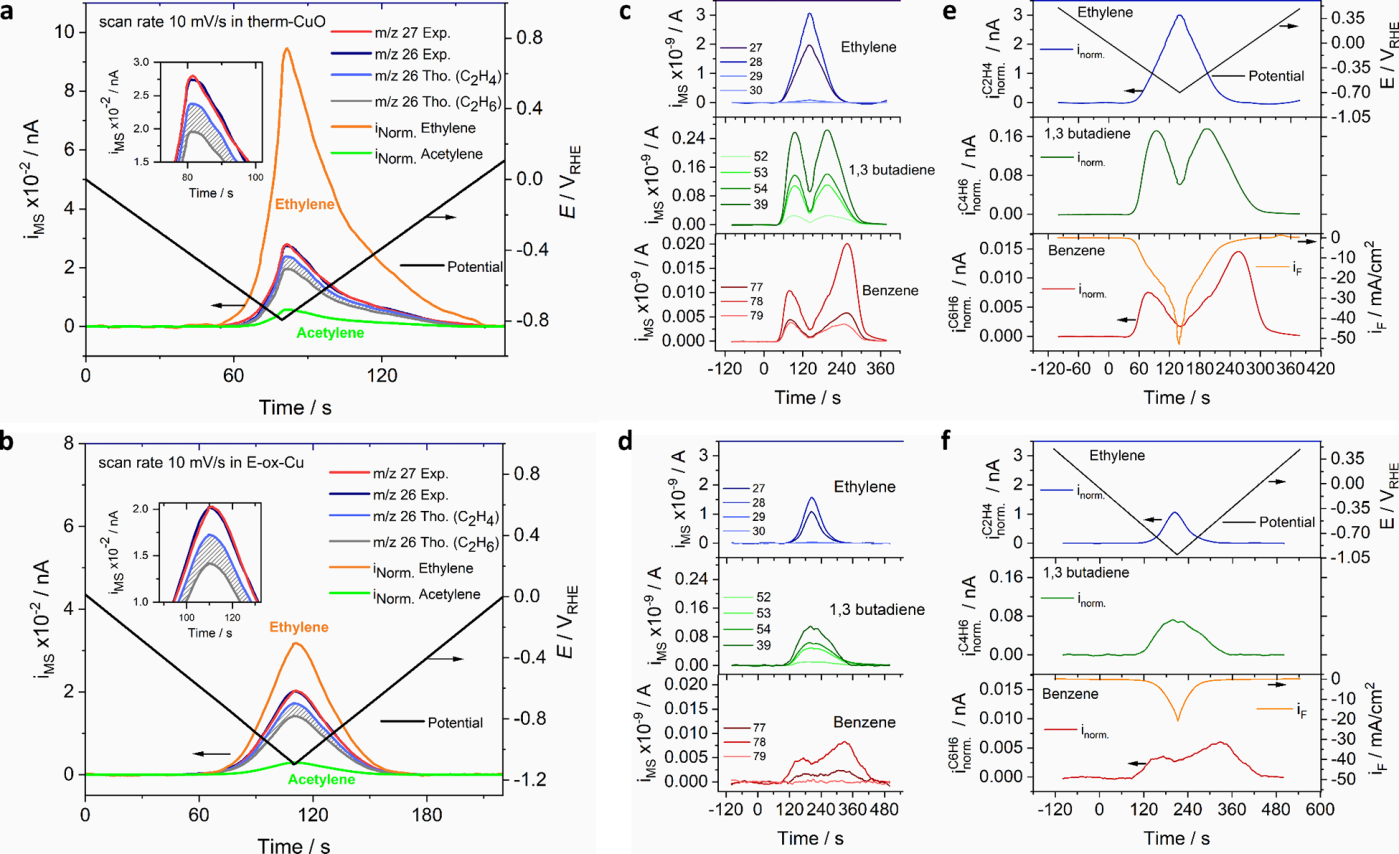
Direct CO_2_ electrolysis to acetylene (C_2_H_2_), electro-hydrodimerization to C_4_ dienes (1,3-butadiene) and cyclo-trimerization ([2 + 2 + 2] electro-cycloaddition) to aromatic benzene (C_6_H_6_): (a and b) Experimentally measured (“Exp”) mass spectrometric cyclic voltammograms (MSCV) in the time domain for *m*/*z* = 26 (dark blue) and *m*/*z* = 27 (red); the theoretically expected (“Tho”) MSCVs for *m*/*z* = 26 assuming either pure ethylene (light blue) or pure ethane (gray) at *m*/*z* = 27 for (a) therm-CuO and (b) E-ox-Cu. Hashed gray area represents expected *m*/*z* = 26 for all ethylene/ethane ratios. Deconvoluted normalized MSCVs (*i*_Norm_) for acetylene (green) and ethylene (orange) are given. Deconvolution is achieved using the *m*/*z* = 26 fragments of ethylene (C_2_H_2_^+^, relative intensity of 55%), ethane (C_2_H_2_^+^, relative intensity of 23%), and acetylene (C_2_H_2_^+^, relative intensity of 100%) and the *m*/*z* = 27 fragment of ethylene (C_2_H_3_^+^, relative intensity of 62%) and of ethane (C_2_H_3_^+^, relative intensity of 33%); a triangular voltammetric cycle was applied starting at +0.54 V_RHE_ to (a) −0.9 V_RHE_ and to (b) −1.1 V_RHE_ at 10 mV/s (black lines, right *y*-axis). Insets: Blow up of the MSCVs near max ion mass currents, partial CO_2_ gas pressure in feed, *p*_CO_2__ = 23 kPa, balance Ar; (c and d) Electrochemical reduction of acetylene to ethylene at *m*/*z* = 27, 28, 29, and 30 (blue lines), acetylene dimerization to 1,3-butadiene (C_4_H_6_) at *m*/*z* = 39, 52, 53, and 54 (green lines), and cyclo-trimerization ([2 + 2 + 2] electro-cycloaddition) to benzene at *m*/*z* = 77, 78, and 79 (red lines) on (c) “therm-CuO” and (d) “E-ox-Cu” catalysts; (e and f) Normalized ion mass currents (*i*_Norm_) of ethylene (blue), butadiene (green), and benzene (red) on (e) “therm-CuO” and (f) “E-ox-Cu” catalysts; the applied potential cycle is shown in the top panels of e and f (black). The measured faradaic current densities are shown in the bottom of panels of e and f (orange). Experimental conditions: 0.1 KOH, ambient temperature, diluted acetylene gas feeds (*P*_acetylene_ = 23 kPa, balance Ar); a voltammetric potential cycle was applied starting at +0.54 V_RHE_ to (c) −0.7 V_RHE_ and to (d) −1.0 V_RHE_ at 5 mV/s (black lines, right *y*-axis). Fragmentation database at https://webbook.nist.gov/. The protocol multiple ion detection was used for detection of all mass spectrum signals.

Numerous previous mechanistic reaction schemes proposed for the CO_2_ reduction process on Cu included a reactive C_2_H_2_ surface intermediate. Yet there has been no report to date of actual direct experimental evidence of the C_2_ alkyne molecule. Owing to the time resolution of capillary DEMS, the detection of shorter-lived intermediates has become feasible. This section presents the first report of acetylene formation during CO_2_ electrolysis on Cu-derived catalysts. The 100% molecular peak (*m*/*z* = 26) of acetylene is strongly convoluted with the *m*/*z* = 26 fragments of ethylene and ethane. Supplementary Figure 12 details our deconvolution strategy in order to extract the acetylene mass signals. Taking peak intensities at *m*/*z* = 30, 28 and 27, the *m*/*z* = 26 peak can be deconvoluted into ethane and ethylene, and the acetylene contribution can thereby be isolated. Note that other C_2_ oxygenates, such as ethanol, have no contribution to *m*/*z* = 26. Figure 4a,b shows the raw DEMS signals at *m*/*z* = 27 (red) and 26 (black) and contrasts them to the theoretically predicted *m*/*z* = 26 ion mass profiles for pure ethylene (blue) and pure ethane (gray) formation. For any ratio of ethane to ethylene the experimental *m*/*z* = 26 would lie within the hatched area. The excess intensity of the actual *m*/*z* = 26 signal suggests the presence of acetylene. The normalized acetylene ion mass profile^31−33,43−45^ is provided in green, while that of ethylene is in orange in Figure 2a,b. While we cannot precisely quantify the faradic acetylene efficiency, we estimate it at this point to a low single-digit percentage. We believe that the high reactivity of acetylene in the presence of hydrogen on Cu has made it impossible to detect it using analytical techniques such as NMR or chromatography. To corroborate our analysis, we investigated the acetylene generation as a function of applied potential sweep rate (Supplementary Figure 13). Faster scan rates of 10–20 mV s^–1^ yielded the largest relative normalized acetylene signals, in line with the notion that acetylene is a highly reacting molecule that is best detected during fast potential scans past its onset potential. We have determined an onset potential of acetylene of −0.55 V_RHE_, −0.67 V_RHE_, and −0.70 V_RHE_ on therm-CuO, E-ox-Cu, and Cu, respectively.

## DEMS Analysis of Electrochemical Acetylene Di- and Cyclo-trimerization to C_4_ and C_6_ Compounds

In the next step, we were interested in exploring the surface electrocatalysis of acetylene on Cu surfaces. To that end, we fed pure acetylene onto the Cu-derived surfaces and recorded the resulting interfacial reaction products using a DEMS system. Figure 4c–f shows the experimental raw and normalized MSCV profiles of all observed ion mass traces during an electrode potential scan from +0.54 V_RHE_ to −0.7 V_RHE_ (Figure 4c,e for therm-CuO) and to −1.0 V_RHE_ (Figure 4 d,f for E-ox-Cu). On either surface, new characteristic ion mass signals were observed, consistent with the formation of ethylene (*m*/*z* = 27, 28, 29, 30); 1,3 butadiene C_4_H_8_ (*m*/*z* = 52, 53, 54, 39); and, interestingly, benzene C_6_H_6_ (*m*/*z* = 77, 78, 79). Tabulated mass fragment spectra aided in and confirmed the identification of the resulting products (Supplementary Figure S14). At open-circuit potential conditions, no acetylene reaction product formation was observed (Supplementary Figure S15). In order to learn more about the acetylene surface reactivity on Cu under electrochemical conditions, we analyzed the chemical and morphological structure of the Cu surface before and after acetylene reaction and compared the results to that of a typical CO_2_ reduction experiment (Supplementary Figures S16–18). Unlike CO_2_ electrolysis, C_2_H_2_ electrolysis left behind a significant coverage of carbon-rich surface deposits, as evidenced in the elemental mapping of Supplementary Figure S17. The massive formation of surface carbon during the reaction of acetylene required the use of dilute acetylene feeds. The onset potentials for the formation of ethylene, butadiene, and benzene are tabulated in Supplementary Table S3. Ethylene formed at more anodic electrode potentials directly from acetylene fills the gap in the mechanistic picture from CO_2_/CO to ethylene as published by Nitopi et al.^5^ and recently updated by Seger et al.^46^ The dimerization and cyclotrimerization products are observed at more cathodic electrode potentials. While the catalytic formation of ethylene and the dimerization to 1,3 butadiene appear to be consistent with a potential-dependent, electrocatalytic proton-electron transfer redox process, the cyclo-trimerization comprises nonelectrochemical surface catalyzed reactions. Their unexpected electrode potential dependence will be discussed in the next section.

## Discussion

We have introduced a capillary differential electrochemical mass spectrometry (DEMS) system that is characterized by rapid capillary flow sampling driven by hydrostatic pressure differences (Figure 1 and Supplementary Figure S4). The design allowed capillary flow conditions with a product time resolution of hundreds of milliseconds. The DEMS product extraction design makes the detection of both gas and liquid products typically involved in CO_2_ electrolysis possible. A dual in-line saturation stack component enabled the accurate control of the gas reactant concentration in the liquid electrolyte feed (Supplementary Figure S6).

## Kinetic Product Onset Potentials

The capillary DEMS system enabled the extraction of the electrochemical onset electrode potentials of a wide set of known and new CO_2_ electrolysis products. The products reported here include major ones such as ethylene, methane, CO, ethanol, and n-propanol but also more rarely addressed intermediates such as acetaldehyde, propionaldehyde, propylene, and acetylene (Figures 2 and 4). The product onset potentials were collected on three distinct Cu-derived electrocatalytic surfaces. The thermal CuO-derived catalytic Cu surface (therm-CuO) showed the most (efficient) anodic onset potentials for all chemical products. The Cu surface that was derived from an electrochemically oxidized CuO_*x*_ surface (E-ox-CuOx) exhibited the second most anodic onset potentials, while the electrochemically reduced metallic Cu surface (Cu) revealed the most cathodic onset potentials. This order can be rationalized based on the initial degree, type, or thickness of oxidic surface species, which, upon in situ electrochemical reduction, resulted in varying abundance or coverages of undercoordinated Cu surface adatoms. Mechanistically complex products that demand excess CO coverages, such as propionaldehyde or propylene, were observed only on OD Cu surfaces. This is consistent with reported higher surface roughness of OD Cu and the associated higher CO binding energy and CO surface coverages compared to smooth metallic Cu (Figure 3).^47,48^

## Mechanistic Implications of Experimental Onset Potentials

**Figure 5:**
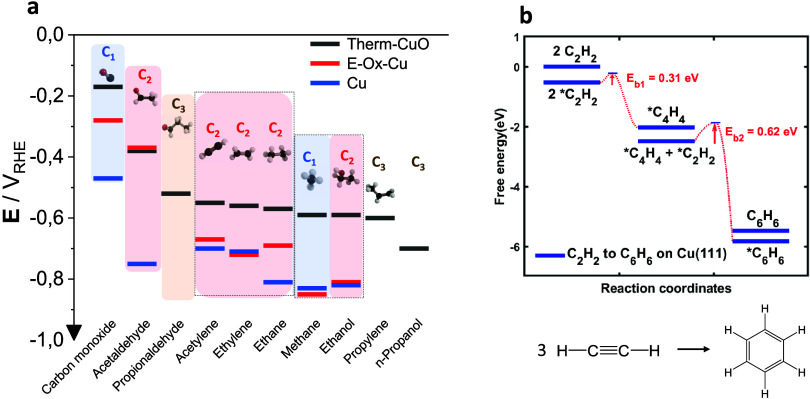
CO2RR product onset potentials and new mechanistic reaction pathways during the CO2RR: (a) Experimental DEMS-derived onset potentials (V_RHE_) of major CO2RR C_1_–C_3_ alkane, alkene, alkyne, and oxygenate products from left to right in decreasing order for the oxide-derived Cu “Therm-CuO” (black) catalyst, the electrochemically oxidized Cu “E-ox-Cu” (red) catalyst, and the metallic polycrystalline Cu “Cu” (blue). C_1_, C_2_, and C_3_ products are marked by cyan, red, and brown color background boxes. Experimental conditions: see Figure 2, cathodic scan rate 2 mV/s. (b) Free energy diagram of acetylene reaction to benzene (3C_2_H_2_ → C_6_H_6_, shown at bottom) with barriers on Cu(111). 2C_2_H_2_ to C_4_H_4_ has a barrier of 0.31 eV, and C_2_H_2_ + C_4_H_4_ to C_6_H_6_ (benzene) has a barrier of 0.62 eV, indicating the acetylene reaction happens spontaneously at room temperature.

To build mechanistic conclusions, we analyzed the reaction kinetics on three different Cu catalysts. The experimentally extracted product onset potentials are plotted in Figure 5a on a common electrode potential scale, ordered by their magnitude observed on the thermal CuO-derived catalysts and colored by carbon number. We believe that these types of onset potential plots offer important mechanistic insights that may complement and support computational mechanistic modeling. A few well-documented product formation sequences are discernible:^5^ Beyond-CO C_1_ and C_2+_ CO_2_ electrolysis starts from molecular CO on the Cu surface that forms as early as −0.18 V_RHE_. CO is the first apparent volatile CO_2_ reduction product on all three surfaces. Subsequently, thermally and electrochemically oxidized Cu-derived catalysts generate acetaldehyde. While the sequential appearance of individual products of similar structure is not unambiguous evidence for a corresponding serial reaction mechanism, it is chemically plausible for acetylene, ethylene, and ethane to occur in exactly this overpotential order at closely spaced electrode potentials on the therm-CuO and OD-Cu surface. The consecutive appearance of propionaldehyde right after acetaldehyde has not been reported before and raises a number of mechanistic questions. It is feasible that it is acetaldehyde that picks up another *C(H)O species to react to the C_3_ aldehyde,^46^ or acetaldehyde and propionaldehyde simply have a common precursor species. On therm-CuO as well as on the other two Cu surfaces, the onset electrode potentials of methane and ethanol appear very closely related. Our current mechanistic understanding of CO_2_ electrolysis places these two products far apart: Ethanol is generally believed to be derived from acetaldehyde/enol intermediates by picking up two hydrogen atoms.^5^ Methane, however, is believed to be limited by a proton coupled electron transfer (PCET) from *C(H)O to *C(H)OH, which subsequently reacts to methyl *CH_3_ surface intermediates and methane.^5^ This is why it is surprising to see the onsets of ethanol and methane being so closely correlated on all three surfaces. In other words, it is unclear why ethanol displays such a significant delay in its onset potential compared to acetaldehyde. Propylene and n-propanol formed at most cathodic onset potentials, likely associated with additional kinetic or steric barriers in the addition of the third carbon atom, perhaps through the surface coupling of acetaldehyde with CO. The offset between n-propanol and propionaldehyde appears surprisingly large, similar to that between acetaldehyde and ethanol.

Beyond acetaldehyde, the onset potential pattern of the electrochemical oxide-derived surface (E-ox-Cu) generally showed closer resemblance to the Cu metallic surface than to therm-CuO. This may be linked to the fast reduction of the electrochemical oxide layers compared to the thick thermally generated CuO layers.

Unlike therm-CuO, beyond CO, metallic Cu first revealed closely spaced acetylene and ethylene before it catalyzed the formation of acetaldehyde and ethane. Just as for therm-CuO, on both E-ox-Cu and metallic Cu, the onsets of methanol and ethanol appeared closely correlated, an observation made earlier on nanostructured Cu catalysts as well. This could indicate the potential existence of an alternative C–C coupling pathway, in which a methyl (*CH_3_) intermediate competitively picks up either an adsorbed *H or a *CHO surface species to yield methane or ethanol. This correlative competition would become active once reactive methyl species have formed on the surface. This is believed to occur at quite cathodic overpotentials, which is why these two products appear together and, in particular, late on metallic Cu and E-ox-Cu. Overall, Figure 5a not only confirms some known but also reveals potentially important novel mechanistic links in the CO_2_ reduction reaction network.

## Acetylene

The observation of acetylene during CO_2_ electrolysis (Figure 4) is an important step forward toward a full clarification of the mechanistic steps of CO_2_ electrolysis. Acetylene offers another missing experimental link in the complex mechanistic CO_2_ reduction network hypothesis. We attribute the fact that acetylene has never been reported before to its high reactivity on metal surfaces, in particular, in the presence of hydrogen surface atoms or hydrogen gas, which favor the electrocatalytic reduction of acetylene to ethylene. Analytical detection hampered by low time resolution, such as gas, liquid, ion chromatography, NMR, or other forms of spectroscopy, is therefore unlikely to produce acetylene signals within detection limits. We believe that, in principle, though short-lived, acetylene can be separated and enriched from CO_2_ electrolyzer outlet streams by means of suitable membrane-based separation schemes. Harvesting acetylene is likely to be more successful near the reactant inlet of Cu-based CO_2_ electrolyzers, where acetylene has not yet had the opportunity to react further. A hydrogen-poor surface environment would prevent initially formed acetylene from being reduced to ethylene. One could envision a structured electrocatalyst surface with intermittent Cu sections, between which acetylene molecules are removed from the feed.

## Benzene

Pyrolytic condensation reactions of acetylene have been known since the work by Berthelot in 1890.^49^ Later, pressurized liquid-phase Nickel-catalyzed acetylene (cyclo)-oligo- and polymerization chemistry was explored in thermo-catalytic reactor environments starting in 1928 and made popular later by the German industrial chemist Walter Reppe, whose name is often associated with acetylene chemistry.^50,51^ Other than Ni, molecular complexes of Fe, Zn, and Cu, and more recently Ti, Pd, and Ir, are known to catalyze (cyclo)oligomerization of alkynes.^52−55^ The formation of benzene from acetylene is believed to follow a highly atom-efficient [2 + 2 + 2] cycloaddition reaction.^51,56,57^ To the best of our knowledge, acetylene oligomerization surface chemistry in electrochemical environments has never been reported before. We rationalize the formation of benzene via a single Cu adatom-catalyzed thermal reaction mechanism that largely follows the [2 + 2 + 2] cycloaddition reaction pathways put forward under high-pressure gas-phase catalytic conditions. We note that the involvement of two, instead of only one, neighboring Cu atoms, which is generally not feasible in the molecular metal complex chemistries, may be possible on our solid Cu surfaces. First, acetylene adds to a surface site M to form a metallacyclopentadienyl species according to
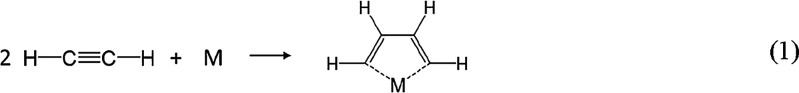
1Subsequently, the complex expands into a metallacycloheptanyl species by adding another C_2_H_2_ moiety according to
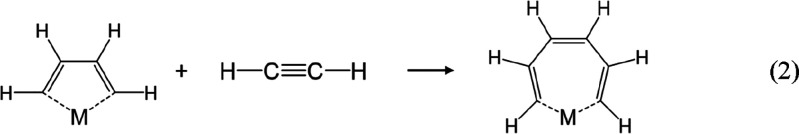
2Before that latter species decomposes into Benzene recovering the bare surface
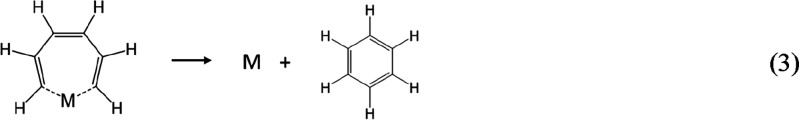
3To better understand the electroless/thermal benzene formation process, free energy diagrams of the acetylene reaction to benzene were calculated with barriers on Cu(111), Figure 5b and Supplementary Figure S19. The reaction step 2 C_2_H_2_ → C_4_H_4_ (eq 1) showed a barrier of 0.31 eV, while the step C_2_H_2_ + C_4_H_4_ → C_6_H_6_ (eq 2) revealed a kinetic barrier of 0.62 eV. These low barriers confirm acetylene reaction happens spontaneously at room temperature on Cu. We further calculated the free energy profile and barrier for acetylene coupling to benzene on Cu(100) and Cu(211) in Supplementary Figure S20. It is seen that due to the square nature of the copper atom arrangement on Cu(100), the barrier for acetylene–acetylene coupling is higher than that of Cu(111). Even steps on Cu(111), that is Cu(211), have slightly higher barriers than Cu(111), while also the desorption of C_6_H_6_ is more difficult. Therefore, we can argue that the formation of benzene mainly happens on Cu(111) instead of on other commonly exposed facets.

Thus, our unexpected electrode potential dependence of the purely chemical (electroless) reaction step to benzene is attributed to the fact that we need to reduce native Cu oxide layers and *OH off the surface of the electrode, which exist under open-circuit potentials. Upon electrochemical reduction of Cu surface oxides to metallic Cu, the acetylene trimerization to benzene reaction can proceed. We note that acetylene is in principle also able to condense to C_6+_ cyclic compounds in the presence of metal catalysts. However, we have not placed focus on a DEMS identification of product species with masses above about *m*/*z* = 80 in this study.

## Carbon and Coke

In typical gas-phase catalytic reaction environments under high pressures and elevated temperatures, acetylene is known to catalytically polymerize, thereby causing often unwanted carbon formation (“coking”).^58−60^ We observed a similar formation of carbon deposits on the surface of the electrified catalytic Cu surface under ambient reaction conditions. The carbon deposits from the dilute acetylene feed are attributed to the large driving force from acetylene to carbon and hydrogen. We investigate the free energy change for coke formation from the reaction
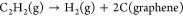
4where C_2_H_2_(g) and H_2_(g) indicate acetylene and hydrogen molecule in gas phase, while 2C(graphene) denotes carbon in the form of graphene. The Δ*G* of this reaction from the DFT calculation is as negative as −2.42 eV. Such a large driving force suggests the facile formation of coke from acetylene under a wide range of scenarios and explains our observations.

## Ethylene and Butadiene

The other two acetylene reduction reaction products, ethylene and 1,3-butadiene, are electrocatalytically generated faradaic products according to
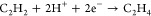
5and
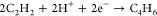
6Whether the molecular reduction of acetylene to butadiene (eq 5) follows reaction 1 prior to reduction or whether two C_2_H_3_ surface intermediates dimerize and get reduced is unclear at this point. While being somewhat obvious, it is interesting to confirm that feeding C_2_H_2_ only allows equal carbon count in products C_2_, C_4_, C_6_, etc. Thus, to achieve an odd carbon count in products there is a need to implement single carbons; that is, in presence of CO_2_/CO one could arrive at C_3_, C_5_, C_7_, etc. products. Whether the combination of an acetylene intermediate and CO is indeed the mechanistic step to C_3_ products has been discussed by Seger et al.^46^

## A Hypothetical Future Electrochemical Process Design for CO_2_ Valorization to Aromatic Compounds

With acetylene being a verified molecular intermediate of CO_2_ electrolysis, we propose the feasibility of a future reactor and reaction process cascade for the reduction of gaseous CO_2_ to aromatic C_6+_ compounds via a highly atom-efficient cycloaddition reaction. First, acetylene is generated and separated from the CO_2_ electrolyte feeds during short contact times at Cu catalysts. This could be realized using porous Cu catalysts that are supported on acetylene-selective membranes. Upon its local generation, acetylene would selectively permeate across the separation membrane based on, for instance, size exclusion principles, driven by pressure differentials. In addition, the membrane-supported porous Cu catalyst layer could be periodically and precisely pulsed cathodically to the onset potential of acetylene to instantaneously generate acetylene near the surface. The subsequent anodic potential pulse would ensure immediate deactivation of the Cu surface in order to prevent hydrogenation or oligomerization of acetylene on the generation side of the membrane. Once permeated, acetylene may pass over another porous Cu catalyst layer applied on the opposite side of the membrane. These could be potentially cylindrical membrane architectures. There, acetylene is reacting to olefinic, aromatic or other more complex chemical compounds. Independently controlled electrode potentials at either Cu catalyst layer would enable a production cascade yielding previously inaccessible reaction products in larger yields than observed to date.

### Conclusions

We used a versatile real-time DEMS system to study the kinetic onset potentials of volatile products during the CO_2_ electrolysis on distinct catalytic Cu-based surfaces. We have presented a previously unachieved comprehensive set of CO_2_ electrolysis products along their onset potentials. Most prominent among them was acetylene, a previously undocumented short-lived alkyne product. The product onset potentials have a mechanistic significance. While sequential or closely spaced onset potentials do not necessarily imply a corresponding close mechanistic location in the reaction network, the onset potentials can greatly support and complement other experimental and computational mechanistic studies. Acetylene as feed in a C_2_H_2_ reduction electrolysis was reported to be reduced, among others, to industrially important C_4_ compounds, such as butadiene, and most importantly was found to react under electrochemical conditions at ambient pressure and temperature to the aromatic C_6_ compound benzene, likely via a [2 + 2 + 2] electroless cycloaddition. A suitable acetylene generation/separation/aromatization process cascade concept was proposed, which would enable the conversion of air-captured CO_2_ into, to date, elusive, yet industrially important e-aromatic compounds. Beyond aqueous electrocatalysis, we are confident that the real-time DEMS system introduced here will benefit fundamental kinetic studies in other disciplines as well, such as bio electrocatalysis or organic electrosynthesis, where time- and potential-resolved tracking of the formation of volatile (organic) compounds is of the essence.

## Data Availability

The DFT results and structural files can also be found at 10.11583/DTU.28378472.

## Supporting Information Available

Material preparation and analytical and synthetic methods and DFT calculation details; supplementary text and discussion; Figures S1–S20 and Tables S1–S3 (PDF)
