# Supplementary material for: Electrochemical CO2 Valorization Pathways and Processes toward C2 to C6 Products: Acetylene, Propylene, Butadiene, and Benzene
Source: ACS Energy Lett. 2025 Apr 29;10(5):2532–42. doi: 10.1021/acsenergylett.5c00467 (PMC12070457; doi:10.1021/acsenergylett.5c00467)
Supplement: Supplementary file 1 — nz5c00467_si_001.pdf [file nz5c00467_si_001.pdf]

Supplementary Information

Electrochemical CO_2 valorization pathways and processes toward C_2 to C_6 products: acetylene, propylene, butadiene, and benzene

Jorge Ferreira de Araújo[1], Jan Rossmeisl[2], Hanqing Yin[3], Xingli Wang[1], Alexander Bagger[4], and Peter Strasser[1]*

[1]Department of Chemistry, Chemical Engineering Division, Technical University Berlin, Straße des 17. Juni 124, 10623, Berlin, Germany.

[2]Department of Chemistry, University of Copenhagen, Copenhagen, Denmark.

[3]Department of Energy, Technical University of Denmark, 2800 Kgs., Lyngby, Denmark.

[4]Department of Physics, Technical University of Denmark, 2800 Kgs., Lyngby, Denmark.

*Corresponding author: Email: pstrasser@tu-berlin.de

This Supplementary Information file includes:

Supplementary Description of Materials and Experimental Methods

Supplementary Text and Discussions

Supplementary Tables S1-S3

Supplementary Figures S1 to S20

1. Experimental details

1.1 Materials, Electrodes, Electrochemical Characterizations

Electrochemical characterization of the carbon dioxide reduction reaction (CO2RR) kinetics was carried out in a customized three-electrode cell setup, referred to as capillary mass spectrometric flow cell ("capillary flow cell"). This customized cell was part of a novel capillary mass spectrometry system described herein. As working electrode, a polished polycrystalline cylindrical copper disk (Ø10 mm x 4 mm, Alfa Aesar PURATRONIC, 99.999% purity) was employed without and with suitable post-preparation treatments (referred to as "Cu", "E-ox-Cu" and "therm-CuO" electrodes, preparation see further below). A customized reversible hydrogen electrode (RHE, bubbling hydrogen at a Pt wire immersed in the bulk electrolyte) was used as reference electrode and a Pt-mesh (Sigma-Aldrich, 99.99% purity) served as the counter electrode downstream from the working electrode.

Polarization, potential control and cyclic voltammetric measurements were done using a Bio-logic SP300 potentiostat. The uncompensated ohmic IR voltage drop was measured and corrected after the cyclic voltammetry (CV) and mass spectrometric cyclic voltammetry (MSCV) experiments using the electrolyte resistance values measured by potentiostatic electrochemical impedance spectroscopy (PEIS).

The customized RHE was prepared in a separated compartment connected to the capillary flow cell via a Luggin-capillary filled with the electrolyte solution of the working electrode compartment. A platinum wire immersed into the H_2 saturated electrolyte served as RHE connection. The RHE compartment was kept under continuous H_2 bubbling and 1 atm of pure hydrogen.

The counter electrode was located in a separate location in the outlet channel of the working electrode compartment. To avoid exchange of reaction products from counter electrode compartment, electrolyte was flown over the counter electrode to the external waste electrolyte reservoir. The contact was made via a 6mm diameter exit flow tube, while a solenoid micropump ensured a continues replacement of electrolyte solution in the capillary cell main compartment. All three electrodes were connected directly to the respective pins of the BIO Logic potentiostat.

1.1.1 Electrode Preparations

Polishing procedure applied to all polycrystalline Cu-based cylinder catalysts

Mechanical / Electrochemical polishing of the as-received copper cylinders was carried out as follows:

(a) First, manual polishing was achieved using three different types of sandpaper (BUEHLER, CarbiMet) starting with lower towards higher grit numbers in order P320, P800 and at last P2500.

(b) Second, machine polishing was performed using diamond paste in order using 9 µm, 3 µm and finally 1 µm particle sizes on grinding/polishing machine (Buehler EcoMet 2500) at a rotation speed of 100 rpm under constant flow of water for 1 minutes.

(c) Third, electropolishing was performed in addition to the mechanical polishing. It is performed in 85% phosphoric acid by applying +4.0 V for 5 minutes between a 10 cm long titanium wire used as anode and a 0.78 cm^2 copper disk surface used as cathode in a meniscus type of setup.

Electrode Characterization

Scanning electron microscopy (SEM) images of the working electrodes were obtained using a JEOL 7401F instrument operated at an acceleration voltage of 10 kV. Energy-dispersive X-ray spectroscopy (EDX) line scan was conducted on the same instrument, utilizing a Bruker Quantax EDX detector at a 15 kV acceleration voltage.

Voltammetric pre-treatments to prepare the "E-ox-Cu" and "Cu" electrodes

The polished Cu disk electrodes were inserted into the DEMS capillary cell and the cell was then sealed, and the electrolyte was saturated with CO_2 gas and, pumped by hydrostatic pressure differences (3 bar gauge in electrolyte reservoir, 2.5 bar in capillary flow cell, 2.2 bar back pressure), through the main cell chamber at few sccm flow rate. Before experimental measurements started, the native thin Cu oxide passivation film that formed on the copper disk surface during handling in air was reduced by applying several potential cycles between −0.2 V_{RHE} and −1.25 V_{RHE} at a sweep rate of 2 mV s^{-1}. To ensure the complete reduction of the Cu surface, we evaluated the mass spectrum signals and the cyclic voltammetric curves. This oxide-free polycrystalline state of the copper disk catalyst was characterized by steady background currents as well as reproducible faradaic HER and CO2RR currents under concomitant reproducible evolution of reduction products (i.e. their mass spectrum signals) over several consecutive scans. We used this test protocol as control and quality check of the initial metallic state of the copper surface before measurements. Figure S1 details X-ray based experiments that showed that the "Cu" catalyst consisted of metallic Cu(0) near the surface, while the "E-ox-Cu" showed signatures of Cu(I) species.

The "Cu" electrode and catalyst

Immediately following the voltammetric pre-treatments and tests of the previous section, the full cyclic voltammogram from -0.2 V_{RHE} down to -1.1 V_{RHE} was started at 2 mV/s, during which MSCVs were recorded, plotted and analyzed.

The "E-ox-Cu" electrode and catalyst

Immediately following the voltammetric pretreatment above, the "E-ox-Cu" electrode was prepared using an additional cyclic voltammetric surface treatment, still in CO_2 -saturated 0.1 M $KHCO_3$ solution (100 kPa CO_2). Starting from -0.2 V_{RHE}, the potential was cycled three times past the Cu oxidation potentials and peaks to +0.8 V_{RHE} and back, using a scan rate of 10 mV/s. The cycling was stopped at +0.8 V_{RHE} during the third cycle. This induced the formation of oxidic Cu redox states at the surface of the electrode. Immediately after, the full cyclic voltammogram down to -1.1 V_{RHE} was started at 2 mV/s during which the MSCVs in the manuscript were recorded and onset potentials were extracted. Subsequent stability studies of the onset potentials involved consecutive potentials cycles between -0.2 V_{RHE} down to -1.1 V_{RHE} for 20 cycles at 5 mV/s.

The "Therm-CuO" catalyst and electrode

After the polishing and electropolishing pretreatment described above, the Cu disk electrode (Ø10 mm x 4 mm) was transferred into a flow furnace and subjected to a thermal reduction and then oxidation treatment (Fig. S1). First the Cu disk underwent a thermal reduction in a mixed gas feed of 5% H_2 and 95% Ar at 200 °C for 30 min. Immediately after, the atmosphere was replaced by a mixture of O_2/Ar (20/80) with temperatures set to 500 °C for 10 min. The color of the Cu disk changed indicating the formation of thick Cu oxide overlayers. This electrode is referred to as the "Therm-CuO" electrode. Figure S1 details X-ray based experiments that showed that the "Therm-CuO" catalyst consisted of Cu(II) species at its surface, most likely CuO.

After the oxidation thermal treatment, the "Therm-CuO" electrode was inserted in the capillary cell and the flow of CO_2 -saturated 0.1 M $KHCO_3$ solution (100 kPa CO_2) was started. Prior to the actual MSCV measurements, one potential cycle from +0.54 V_{RHE} to –0.9 V_{RHE} at 2 mV/s to reduce the Cu oxide at the surface and to form a stabilized oxide-derived catalyst surface. Subsequent stability studies of the onset potentials involved consecutive potentials cycles between -0.2 V_{RHE} down to -0.9 V_{RHE} for 20 cycles at 5 mV/s.

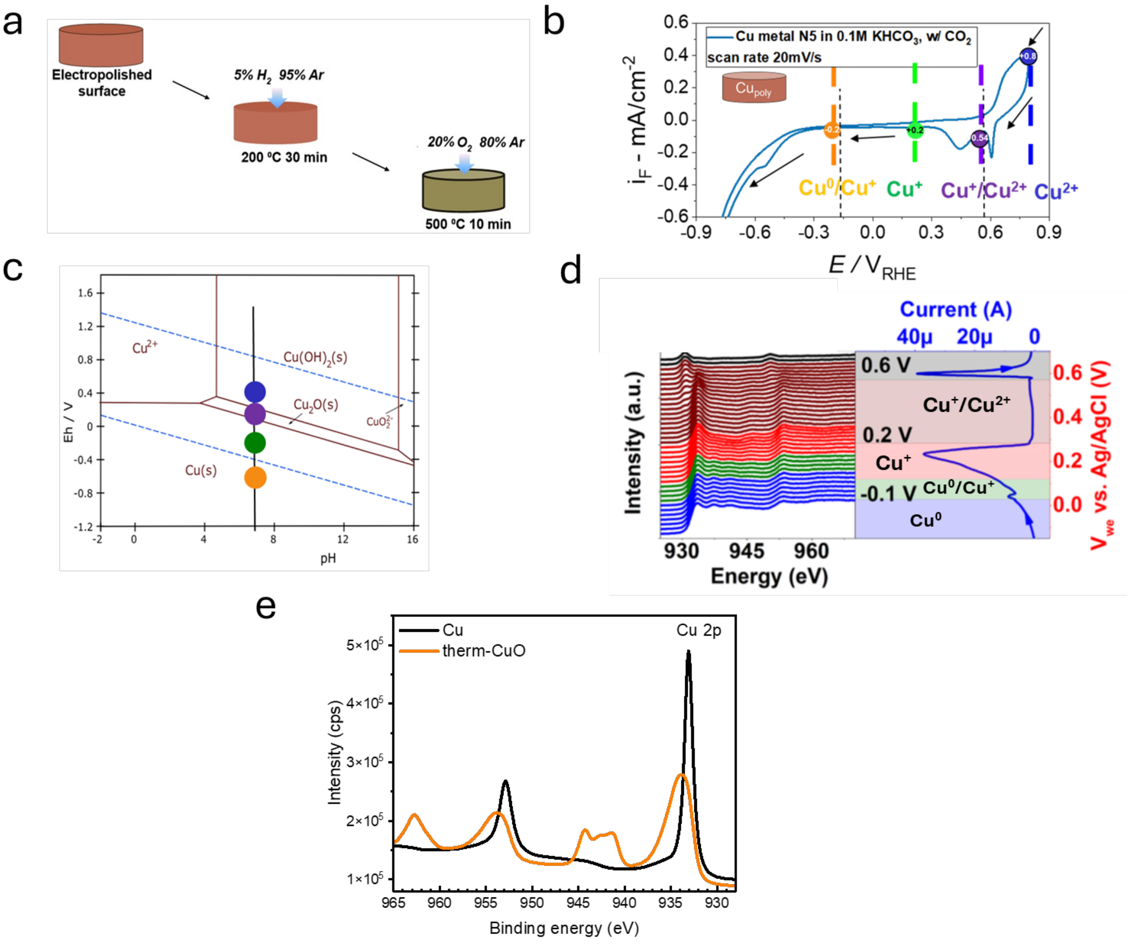

Supplementary Figure S1: Preparation and Characterization of the Cu electrode surfaces used: **a** consecutive thermal treatments of an electropolished polycrystalline copper disk to prepare the oxidic Cu(II)O surface referred to as **"therm-CuO"**; Feed gas compositions (100 sccm total volumetric flow rate) flown through a quartz gas flow tube furnace, as well as temperature and temperature hold times are shown. **b** Cyclic voltammetric profile of a polished polycrystalline metallic Cu film surface. Vertical dashed lines show selected electrode potentials and their corresponding Cu redox states near the surface. **c** Thermodynamic Pourbaix Diagram of Cu where the selected electrode potentials of partial Figure a are marked. **d** In-situ X-ray absorption spectra at the Cu L_3 edge as function of applied electrode potential and the corresponding dominant Cu redox states. The voltammetric pretreatment of the **"E-ox-Cu"** surface at +0.8 V_{RHE} in pH ~7 (~ 0.2 V_{AgCl}) results in the dominant formation of Cu(I) species near the electrode surface (Reprinted (adapted) with permission from Velasco-Vélez et al., ACS Sustainable Chemistry & Engineering, 2019. Copyright © 2019 American Chemical Society.). **e** X-ray

photoemission spectra in the Cu2p range of the metallic Cu **(“Cu”)** and the thermally oxidized CuO electrode (“**therm-CuO”**).

1.1.2 Differential Electrochemical Mass Spectrometry (DEMS) – Set up and operation of a novel DEMS set up

DEMS is an chemical analysis technique that allows a time-resolved identification and quantification of both gaseous and volatile liquid products generated at electrocatalytic (electrified) interfaces during faradaic charge transfer surface reactions involving bond breaking and bond making. The electrochemistry is performed in a DEMS in-situ cell with defined and time-stable electrolyte flow regime. Pressure differentials must be carefully controlled to achieve stable and meaningful mass spectrometric background currents. The major advantage of such a system is the time-resolved continuous analytical quantification of the production rates, onset potentials, and integrated molar amounts of all volatile gaseous and liquid reaction products. The dynamic DEMS analysis enables the quasi-instantaneous measurement of potential-dependent product formation rates.

The Mass spectrometric analyzer system used in this work was composed of two consecutive vacuum sections with one turbomolecular pump (Pfeiffer TMP HiPace 80) mounted directly in each section (differential pumping). The two vacuum sections are connected by an internal pin hole of 6 mm diameter. While in operating, the pressure across the sections drops from 10^{-3} mbar in the first chamber (low vacuum at extraction stage) to 10^{-6} mbar range in the analyzer QMS chamber (high vacuum at the analyzer stage), resulting in differentially pumped system. The experimentally measured ion mass current intensity of a chemical compound j, $i_{MS,j}$, at a given mass to charge (m/z) ratio is linked to the partial pressure of the molecular compound j, p_j, at the ion source via a sensitivity factor K_j according to [1-3]

$$i_{MS,j} = p_j\, K_j \quad [A] \tag{S1}$$

where K_j includes the molecular “ionization probability” at a given electron energy (here 70 eV) in the ion source, the “fragmentation ratio” (ratio of m/z fragment intensity to total intensity of all fragments), and the “transmission factor” describing the ion collection efficiency of the detector across the quadrupole mass filter. The partial pressure of product j, p_j, in turn, depends on the molar product flow from the electrochemical cell across the capillary extractor into the ion source, $\dot{n}_j$, and the volume pump rate, S, of the vacuum chamber as[4]

$$p_j = \mathrm{R\,T}\,\frac{\dot{n}_j}{\mathrm{S}}\ [\mathrm{mbar}]. \tag{S2}$$

Finally, $\dot{n}_j$ depends linearly on the total faradic interfacial current, I, at the working electrode through another sensitivity factor, K_j', that includes the specific DEMS cell configuration, its product collection efficiency, the faradaic efficiency of product j, and all other operation, flow, and materials parameters of the electrochemistry/vacuum interface.[4, 5] While, in principle, K_j' is experimentally accessible, if suitable catalysts with known product selectivity are available and $\dot{n}_j$ can be determined, practical estimation of K_j' is often difficult and actually not necessary. Together with the equations above, one obtains the relation

$$i_{MS,j} = \mathrm{R\,T}\frac{K_j'}{\mathrm{s}}K_j\,I \qquad \text{(S3)}$$

stating that the faradaic electrochemical current, I, is proportional to the observed ion mass current. If K_j' values are not readily available, K_j -normalized mass current intensities ($i_{MS,j}\,/K_j$) provide partial pressures of volatile product molecules at and, equivalently, molar product flows of volatile product molecules into the ion source of the mass spectrometer. In many cases, absolute K_j values of individual compounds are not accurately known, as they depend on detailed ion source, quadrupole system and operation conditions. However, K_j values of compound j that were normalized with respect to a given reference species, typically N_2, are tabulated in the literature. They are referred to as relative sensitivity factors, RSF_j , where $RSF_j = K_j/K_{N2}$.[6-8] RSF-normalized ion mass currents of the 100% main peak fragment of compound j are referred to as i_{Norm} in some MSCV plots in the present study. The integral of i_{Norm} of a given species is a measure of a molar amount of the compound. Ratios of integral i_{Norm} values (integrated over time) can thus be used to estimate relative compound production rate efficiencies. The physical meaning of the RSF_j values is discussed below.

In the present study, the mass spectrometric measurements were performed with $1\text{x}10^{-6}$ mbar pressure at the PrismaTM quadrupole mass spectrometer analyzer of the QMS 220 device supplied by Pfeiffer-Vacuum Inc. The ionization process was achieved using a gas-tight ion source with two tungsten (W) filaments (filament current of 1 mA) at temperature ~44 °C. The quadrupole filter consists of four stainless steel rods with length of 100 mm and each one with 6 mm in diameter, all together with an optimized for mass range 1–100 amu. As detector, a continuous secondary electron multiplier (C-SEM) with optimized voltage of 1050 V (three orders magnitude higher mass signal intensity compared to the Faraday cup) is used.

The completed MS sensor setup is placed in the vacuum chamber with an internal diameter of 600 mm. Mass spectrometric data is collected with Quadera® software. The measurement parameters

using detection mode multiple ion detectors (MID mode) generally involve a dwell time of 50 ms. All mass signals presented in this work were baseline subtracted.

The coupling of electrochemistry with MS has led to a variety of flow cell designs and pumping designs that constitute a technique called Differential Electrochemical Mass Spectrometry (DEMS), where an electrochemical electrolyte flow supports the diffusion process of reaction products to the liquid/vacuum interface in order to maintain *plug flow conditions* for time-resolved detection of products. The undesired back mixing of interfacial reaction products is there by minimized. The design of flow systems promotes an enhanced mass transport mechanism, resulting in fast product detection at millisecond range (real-time analysis).

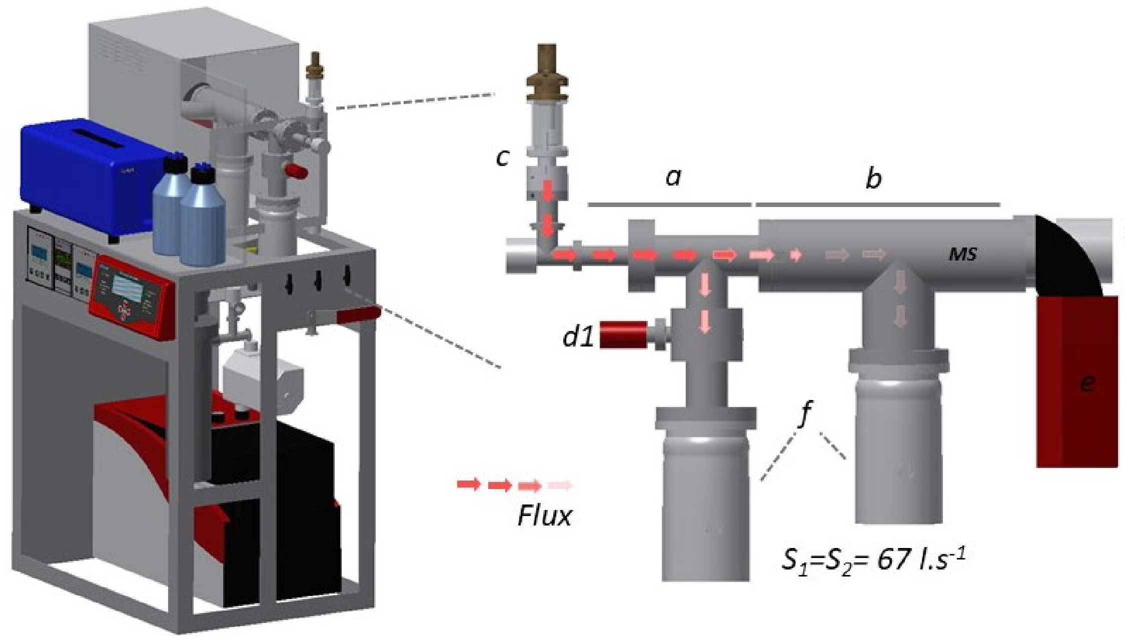

Supplementary Figure S2. Schematic customized Differential electrochemical Mass Spectrometry (DEMS) setup used in this study. The system features two coupled differentially pumped chambers using two Turbo molecular pumps (TMP) interfacing to a new custom-made electrochemical capillary DEMS flow cell. a) first stage vacuum chamber, b) second stage vacuum chamber and quadrupole mass spectrum (QMS) with gas tight ion source, c) capillary flow cell, d1) and d2) pressure sensors, e) electronics QME, f) turbo molecular pumps (TMPs). S1 and S2 represent the resulting pump speed. The red arrows indicate the “Flux”, that is the flow path of product gases inside the system.

1.1.3 The novel capillary flow cell sampling system and its basic principles of operation – Extracting accurate product onset potentials

The novelty of the current DEMS set up is the collecting capillary near the catalytic interface that partially collects the products generated at the electrode surface continuously (Fig. S3 and Fig.S4A). Unlike earlier approaches, the capillary top is deployed near the reactive electrified interface without any porous membrane at the capillary inlet, in order to maximize the collection efficiency and minimize the response time of the MS detection. Operated under plug flow conditions, the capillary sampling loop ensures that the local and temporal concentration gradients of sampled products at the electrified interface are fully preserved during potential scanning up to the product molecule transfer across the membrane interface. Plug flow conditions ensue from the high capillary electrolyte flow velocity inside the capillary tube with maximum speeds > 0.5 m/s (see Fig. 1, Fig. S3 and Fig.S4). These electrolyte capillary flow velocities were determined based on liquid collection experiments and were confirmed by CFD calculations. The low back diffusion and low residence time inside the capillary is the reason for minimal dilution or other undesired chromatographic mixing effects. On the other hand, the fast capillary flow guarantees a nearly simultaneous MS detection of each independent compound and a preservation of the order of their generation at the interface. The plug flow conditions across the capillary and across the porous membrane are a prerequisite for *accurate time-resolved determination of onset potentials for each product*. The capillary is operated under concomitant convective inflow and balanced outflow of electrolyte. Fresh saturated electrolyte is flown over the catalytic interface to minimize transport gradients and local depletion. To balance the internal volume of electrolyte, the electrolyte outflow is controlled by hydrostatic pressure differentials to maintain a constant bulk electrolyte volume of ~ 50 mL inside the capillary cell.

The sampled capillary electrolyte flow is injected into the extractor, that is a thin layer compartment with fixed volume on top of a nonporous PTFE membrane interfacing electrolyte and vacuum (Fig.S4B). At the capillary inlet, the electrolyte volume between capillary inlet the catalytic surface of the working electrode (WE) is exchanged around 377 times per second based on the determined high capillary flow velocity resulting in a capillary outflow rate of 2 μl/s. This process allows for a significant rising of MS sensitivity for short-lived reactive species or highly diffusive compounds, as needed for the detection of acetylene and other short-lived intermediates. The PTFE membrane area is about 10,000 time larger than the capillary inlet area, which ensures high detection sensitivity, as volatile products are effectively and quantitatively transferred across the membrane into the spectrometer vacuum.

The membrane interface generates an ideal and homogenous liquid contact over the porous surface (Fig.S4C). The hydrostatic pressure-driven flow of vaporized species is a result of the gradual increase of vacuum pressure under the membrane interface throughout the frit porous medium. The constant hydrostatic pressure of electrolyte on top of the porous membrane and the vacuum pressure causes a continuous liquid contact and, consequently, an effective convective flow of vaporized species through

the vacuum regime. The drop of pressure generated with a differential pumping system is essential for high throughout pumping for volatile compounds and water vapor into the high vacuum regime. The vaporized analytes elevate continuous the vacuum pressure up to $1x10^{-3}$ mbar, without any additional dilution from carrier gases before entering the MS.

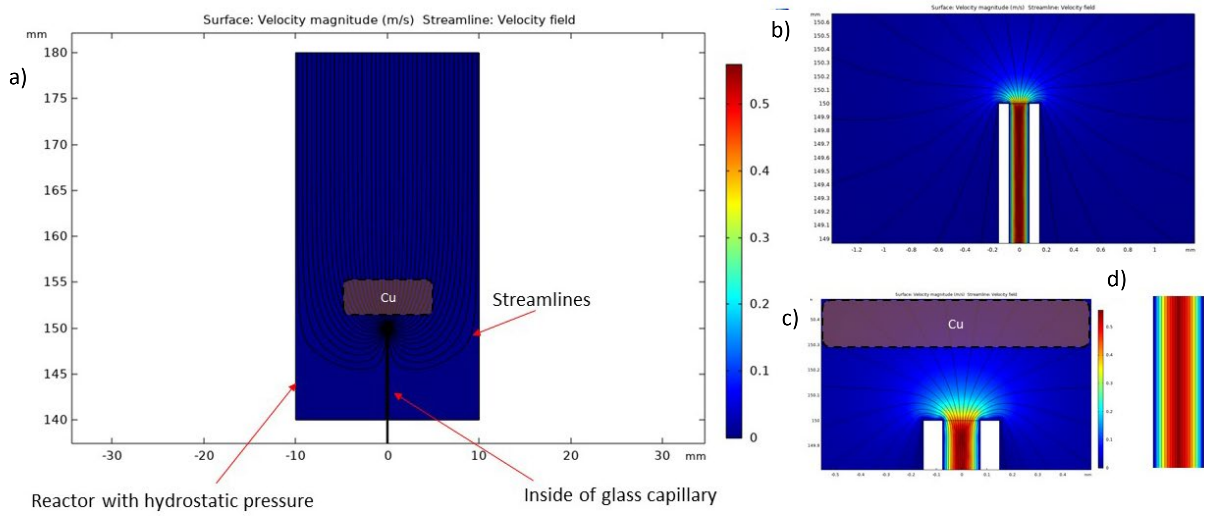

Supplementary Figure S3. Electrolyte flow velocity simulations inside the DEMS capillary cell: **a)** Simulations of the electrolyte velocity distribution (color coded in m/s) around the projection of a disk shaped Cu electrode (grey) outside and inside a capillary located below the Cu surface. This configuration is to resembles the electrolyte volume of the Capillary flow cell used in this study. Color coding indicates the velocity magnitude (warmer color is faster). **b)** and **c)** Blow-ups of a portion of Figure a), showing the velocity profiles at and near the inlet of the capillary in more detail. **d)** Electrolyte velocity gradient inside the capillary sampling the products to the liquid/vacuum extractor interface. Simulation study was done using CDF COMSOL Multiphysics software with following parameters: capillary cell head space pressure 2.5 bar; Backpressure of 2.2 bar; Outlet capillary flow rate 2 μl/s.

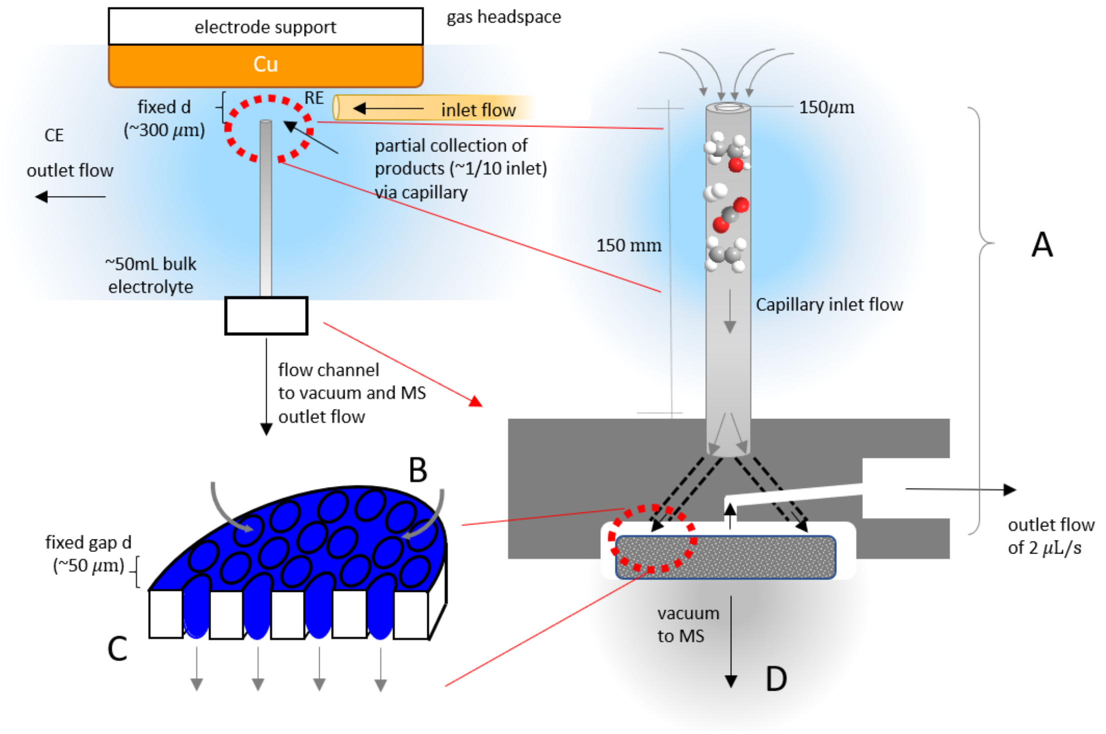

Supplementary Figure S4: Schematic overview of the design components of the Capillary Differential Electrochemical Mass Spectrometric flow cell with a 50 ml bulk electrolyte chamber. A) detailed schematic view of the capillary dimensions (150 μm diameter) and the link to the gas/liquid extractor with outlet flow of about 1/10 of the bulk inlet flow rate, The distance of the capillary from the Cu catalytic interface is around 300 μm; B) and C) schematic design of the hydrophobic PTFE membrane separating the liquid and vacuum regions C) and D).

1.1.4 DEMS system flow diagram including the electrolyte-gas saturation components

Fig. S5 shows the schematic flow of liquids and gases at the gas/liquid saturation components as well as the hydrostatic pressure controls of the overall DEMS system. The primary objective of the liquid flow system is to maintain a consistent and uninterrupted flow of electrolyte, free from pulsations or bubble formation, while ensuring it remains saturated with reactant gas or gas mixtures. This is essential for achieving accurate measurements and efficient electrochemical reactions. Hydrostatic pressure differentials regulate liquid flow rates throughout the system, maintaining a constant flow. Additionally, the back pressure valve ensures stable pressure throughout the entire fluidic system, including the electrochemical cell and product extraction at the vacuum interface. This stability contributes to consistent liquid pressure and reliable mass spectrometry baselines. The principles of the liquid flow system are achieved by directly injecting the electrolyte from a hydrostatically pressurized reservoir into the electrochemical cell. Before reaching the cell, the electrolyte is saturated using an in-line gas/liquid saturation stack (dual in-line saturation stack). The saturation level of the electrolyte with gas can be precisely adjusted to the desired concentration, either by achieving maximum saturation at a 1:1 liquid-to-gas pressure ratio or by diluting the pre-saturated electrolyte with a non-saturated electrolyte solution. The use of a specifically designed saturator enables continuous saturation, precise control of gas concentration in the solution, and minimizes degassing with minimal bubble formation. This system offers the significant advantage of in-flow saturation. CO_2 is passed over a porous, hydrophobic PTFE membrane, ensuring efficient gas transfer in accordance with Henry's Law. The saturated electrolyte, maintained at constant concentration levels, is injected near the electrode and capillary interface to provide a stable mass spectrometry baseline signal.

The liquid flow system consists of several components working in tandem to ensure efficient operation and precise sampling. The core component is the capillary flow cell (1), where reaction products are collected using a small capillary tube, using an internal elevated pressure (2). The tube is positioned approximately 300 μm from the electrode surface with the aid of a webcam microscope (14) for precise alignment. The capillary tube position is angled at 45° relative to the fresh electrolyte feed (3) tube to optimize flow dynamics and sampling efficiency. The flow cell has a capacity of 100 mL, accommodating a platinum mesh electrode placed near the working electrode. These electrodes are externally connected to a potentiostat device (4) for accurate electrochemical measurements. A syringe pump (5) is employed for parallel electrolyte feeding, allowing precise control over electrolyte dilution by adjusting the flow rate ratio. A selective valve (6) facilitates the syringe's refill with fresh electrolyte. To avoid premature deactivation of the copper electrode during experiments with acetylene, a diluted electrolyte flow was used via adjustment of two in desired ratio. The entire fluid system is housed in a gas-tight box (7) to isolate it from potential gas leaks and ensure safety. The electrolyte is stored in a glass laboratory reservoir (GL45) (9), graded for elevated pressure. A pressure regulator and gas flow control system (11) are connected to the supply gas bottles to maintain consistent pressure for the

hydrostatic system. Reservoir pressure is further managed using a leak valve (12) to vent the reservoir atmosphere as needed. Electrolyte saturation with specific gases is performed before entering the capillary flow cell using a dual in-line saturation stack (13). This component ensures efficient, uninterrupted gas saturation of the electrolyte, achieving high saturation efficiency critical for the experiment.

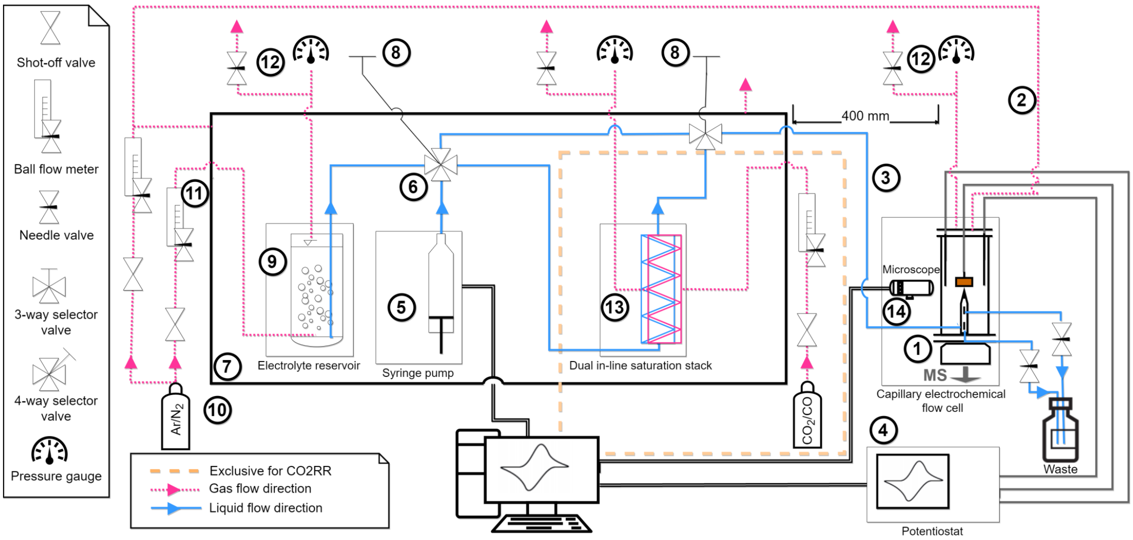

Supplementary Figure S5: Schematic overview of the overall DEMS liquid electrolyte and saturation gas flows in and out of the capillary DEMS flow cell (1). Components include feedthroughs and flow lines of gases, e.g. in the head space of the capillary cell (2) (pink dashed lines), liquids (3) (blue solid lines), and electrical connections (4) (gray solid lines). The syringe pump (5) and a 4-way ball selector valve (6) are located within a hermetically air-sealed glove box (7). Valves rotators (8) can be operated from outside the box manually. Electrolyte reservoirs (9) are also located inside the glove box and are supplied by various gas lines (10) fitted with ball flow meters (11). The electrolyte reservoir's and capillary cell's internal pressure are regulated and controlled by combination of a needle valve and pairs of pressure gauges (12) above the reservoir and in head space of the cell. The dual in-line saturation stack (13) operates via continuous liquid flow from the syringe pump against pressurized CO_2 gas. Syringe and distance control video microscope (14) are operated remotely via a computer.

Highly efficient saturation is accomplished with stacking of several blocks of membrane and electrolyte flow layer resulting in a high ratio of CO_2 concentration to total volume of gas used. This setup was advantage for experiments that using rather expensive isotopes labelled gas.

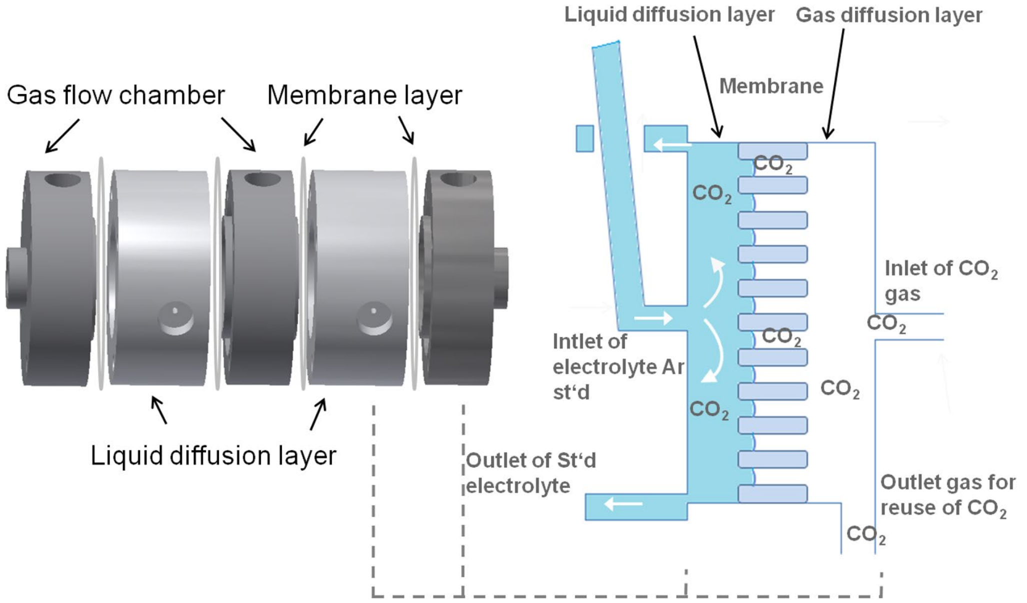

Supplementary Figure S6: left: Schematic diagram of the *dual in-line saturation stack* setup; liquid flow channels (light grey) interface via PTFE membranes to gas (e.g. CO_2) flow compartments (dark grey). **Right**: The gas saturation process is shown in cross section view across the PTFE membrane in the schematic illustration on right.

1.1.5 The Quadrupole Mass spectrometer and principles of operation in the current DEMS configuration

The ionization process is designed to be a compromise between the electron energy and number of ions produced, such that between 10 to 20 eV is typically transferred to the molecule to be ionized. Depending on the mixture of gas to be analyzed, the calibration of the ion source can be adjusted to accelerate the electron at ideal compound ionization energy (eV). This ionization technique produces almost exclusively positively charged ions. Only in rare exceptional cases, parts or all of the molecular ions break and fragment into neutral atoms, and a limited range of substances may even generate negative radical-anions (M•−). A typical electron impact ionization at 70 eV in a high vacuum is applied for mass spectrometry in this study. The level of fragmentation of molecule M into fragments A, B depends on primary structure, electron energy and ion source temperature according to the following equation:

$$M + e^- \rightarrow M^{\cdot +} + 2e^- \rightarrow A^+ + B^+ \qquad (S4)$$

This fragmentation pattern is used primally for the identification of unknown substances or for quantification analysis of a mixture of gases. The resulting fragmentation patterns for given electron impact are highly specific and universal for each molecule and can therefore be used to deconvolute the presence of minute quantities of compounds in case of overlapping ion mass peaks. This is known as a molecule "fingerprinting" and deconvolution. Supplementary Table 1 below shows some of the target compounds studied in this study (leftmost column), with their major and minor peaks and relative sensitivity factors, RSF (see discussion in 1.1.8 below), where available. Databases with fragmentation patterns of numerous molecules are available at http://webbook.nist.gov/chemistry/mw-ser.html.

Supplementary Table S1: Selected compounds and reaction products relevant to the catalytic reaction and conditions studied in the present work along with the relative intensity of their major mass spectrometric fragments and their relative sensitivity factors (RSF).[6] The data apply to electron impact ionization at 70 eV. The relative sensitivity (RSF) represents a combined probability of ionization, fragmentation, and Quadrupole transmission of the 100% intensity peak species.

*1. Relative Sensitivity and RS Measurement of Gases - Application Note 282. Hiden Analytical: 2021 (Accessed 20.1.2022)

Compound		Main peak	Intensity	Next peak	Intensity	Next peak	Intensity	Next peak	Intensity	Next peak	Intensity	RSF*
Formula	Name	m/z	%	m/z	%	m/z	%	m/z	%	m/z	%	
H_2	*Hydrogen*	2	100	-	-	-	-	-	-	-	-	0.84
CH_4	*Methane*	16	100	15	85	14	19	13	8	12	2	0.75
H_2O	*Water*	18	100	17	23	16	2	-	-	-	-	0.9
C_2H_2	*Acetylene*	26	100	25	20	24	6	13	6	-	-	1.28
C_2H_4	*Ethylene*	28	100	27	65	26	55	25	12	-	-	1.02
CO	*Carbon monoxide*	28	100	12	5	16	1	-	-	-	-	0.98
N_2	*Nitrogen*	28	100	14	7	29	1	-	-	-	-	1,00
C_2H_6	*Ethane*	28	100	27	33	30	26	26	23	29	22	1.35
C_2H_4O	*Acetaldehyde*	29	100	44	46	43	27	42	9	26	5.5	1.0
CH_3OH	*Methanol*	31	100	32	67	29	65	28	6	-	-	1,80
C_2H_5OH	*Ethanol*	31	100	45	51	29	30	27	24	-	-	1.76
O_2	*Oxygen*	32	100	16	11	-	-	-	-	-	-	0,86
Ar	*Argon*	40	100	20	11	-	-	-	-	-	-	1,20
C_3H_6O	*Acetone*	43	100	58	33	15	20	42	9.6	27	7	3,60
CO_2	*Carbon dioxide*	44	100	28	12	16	16	12	7	22	3	1.17
C_3H_6O	*Propionaldehyde*	58	100	29	88	28	58	27	47	57	30	n.a.
C_3H_8O	*n-propanol*	31	100	29	18	27	16	59	11	60	7	n.a.
C_4H_6	*1,3-Butadiene*	39	100	54	95	53	71	50	27	51	25	1.5
C_6H_6	*Benzene*	78	100	77	28	51	22	50	21	52	19	1.38
C_xH_y	*Rotary pump oils*	57	100	55	73	43	73	41	33	-	-	1,00

1.1.6 Specific DEMS Set up and experimental parameters for the present CO2RR experiments

In the present CO2RR DEMS experiments, volatile chemical products were recorded at the selective mass/charge channel m/z= 2, 14, 15, 16, 17, 18, 20, 22, 22.5, 25, 26, 27, 28, 29, 30, 31, 32, 40, 41, 42, 43, 44, 45 and 46 using the Quadera MID mode during the applied CV scans.

The flow system in the periphery of the DEMS capillary flow cell uses three independent liquid flow streams. A constant electrolyte flow of 20 µl s^{-1} originating from the hydrostatic pressure electrolyte vessel passes through a saturation stack and becomes a CO_2-enriched solution, before entering the electrochemical DEMS cell. The sampling flow through the capillary is maintained constant at 2 µl s^{-1} controlled by the hydrostatic overpressure inside the DEMS capillary cell of about 150 kPa versus ambient pressure (2.5 bar absolute pressure, see Fig S3). This differential is controlled by a high precision pressure gauge. The counter electrode outlet electrolyte flow from the DEMS cell is kept constant at 18 µl s^{-1} with an adjustable speed solenoid pump. The main cell body is made from glass, with both ends made leak-tight using Kwik-Flange™ ISO KF and the internal cell tubes and WE holder being made of PEEK material.

1.1.7 Mass signal deconvolutions of products used in this study

A deconvolution analysis based on multiple peaks of the fragmentation patterns was applied to ion mass signals composed of and convoluted by the superposition of more than one fragment species. For instance, the contribution of electrocatalytically formed CO to the experimental ion mass signal at m/z= 28 (CO+) can be deconvoluted and accurately extracted from the background corrected experimental signal by correcting for the fragment contributions of ethylene and carbon dioxide, which, in turn, can be extracted from uniquely characteristic ion mass fragments, such as m/z= 27 ($C_2H_3^{\cdot+}$) or m/z= 22 (CO_2^{++}) for ethylene and carbon dioxide, respectively. Product fragmentation patterns were experimentally determined using pure gas feeds under experimental conditions, or else, they were taken from standard tables. The tabulated relative intensities of two or more fragments and their changes help deconvolute consumption and generation of reactant species. Individual product contributions to various ion mass signals, $I_{m/z}$, was achieved using product fragment relative intensity (see Table S1). A few selected masses and the associated products are discussed in the following:

Mass 30 (Ethane)

The experimentally measured signal at m/z= 30 ($I_{m/z\ 30\ (experimental)}$) can be safely attributed exclusively to ethane, $I_{m/z\ 30\ (ethane)}$ (see fragmentation in Table S1 or in Figure S12b). This is because the contributions of ethylene, $I_{m/z\ 30\ (ethylene)}$, acetaldehyde, $I_{m/z\ 30\ (acetaldehyde)}$, and CO, $I_{m/z\ 30\ (CO)}$ based on singly or multiply ^{13}C or ^{18}O isotope-labelled fragments is very small (0.01 - 1 %). From $I_{m/z\ 30\ (ethane)}$, $I_{m/z\ 27\ (ethane)}$ and $I_{m/z\ 26}$

$_{(ethane)}$ can be calculated using the relative fragmentation intensities (Table S1, also Figure S12b) 0.79 and 1.13, respectively. We note that outside m/z=30, however, the ion mass signal contributions of ethane during CO2RR, $I_{m/z\ 27\ (ethane)}$ and $I_{m/z\ 26\ (ethane)}$, are generally very weak based its chemical selectivity and faradaic efficiency.

$$I_{m/z\ 30\ (ethane)} = I_{m/z\ 30\ (experimental)} = I_{m/z\ 27\ (ethane)} \times 0.79 = I_{m/z\ 26\ (ethane)} \times 1.13 \qquad (S5)$$

Mass 29 (Acetaldehyde)

Attributing $I_{m/z\ 30\ (experimental)}$ to ethane, the ion mass at m/z = 29 can be evaluated and attributed to acetaldehyde:

$$I_{m/z\ 29\ (acetaldehyde)} = I_{m/z\ 29\ (experimental)} - I_{m/z\ 30\ (ethane)} \times 0.85 - I_{m/z\ 27\ (ethylene)} \times 0.01 - I_{m/z\ 28\ (CO)} \times 0.01 \qquad (S6)$$

Mass 28 (CO, ethylene, ethane, CO2)

The ion mass signal at m/z = 28 is high due to the molecular CO reaction product ($I_{m/z\ 28\ (CO)}$) and the CO fragment formed by CO_2 fragmentation ($I_{m/z\ 28\ (CO2)}$). The contribution of CO_2 fragmentation at m/z = 28 can be deconvoluted thanks to the doubly ionized CO_2^{++} m/z = 22. Note that CO_2 is consumed during the reaction, which makes the background normalized ion mass intensities, $I_{m/z\ 22\ (CO2)}$ and $I_{m/z\ 28\ (CO2)}$, negative numbers, i.e. they lower the experimentally observable signal intensity at m/z = 28. The signal at m/z = 22 can be exclusively attributed to the doubly ionized CO_2, which can be used to correct for CO_2 contributions at m/z = 28:

$$I_{m/z\ 28\ (CO2)} = I_{m/z\ 22\ (CO2)} \times 4 \qquad (S7)$$

$$I_{m/z\ 28\ (ethane)} = I_{m/z\ 30\ (ethane)} \times 3.84 \qquad (S8)$$

$$I_{m/z\ 28\ (ethylene)} = I_{m/z\ 27\ (ethylene)} \times 1.61 \qquad (S9)$$

$$I_{m/z\ 28\ (CO)} = I_{m/z\ 28\ (experimental)} - I_{m/z\ 28\ (CO2)} - I_{m/z\ 28\ (ethylene)} - I_{m/z\ 28\ (ethane)} \qquad (S10)$$

Mass 27 (Ethylene)

The ion mass signal m/z = 27 has primary contributions from ethylene and ethane, with that of ethane being very small. Background interference effects due to high signals at m/z = 28 (extended signal tails) can affect the accuracy of the evaluation of the signals at m/z = 27. This is why a dilute CO_2 partial pressure was used in part of the experiments (p_{CO2} = 23 kPa). Contributions of ethane can be deconvoluted from those of ethylene using the following procedure:

$$I_{m/z\ 27\ (ethane)} = I_{m/z\ 30\ (ethane)} \times 1.27 \qquad (S11)$$

$$I_{m/z\ 27\ (ethylene)} = I_{m/z\ 27\ (experimental)} - I_{m/z\ 27\ (ethane)} \qquad (S12)$$

Mass 26 (Ethylene, Ethane and Acetylene)

The detection of acetylene is deduced from the m/z= 26 ion mass current signal, which is the 100% main molecule signal. To deconvolute the m/z= 26 ion mass current signal and extract the acetylene contribution the following procedure was applied (see Fig. 4a,b and Fig. S13 and Fig. S12):
First, the experimentally measured m/z (C2H3+) =27 (red in Figure 4a,b Figure S13) and m/z (C2H2+) = 26 (black in Figure 4a,b Figure S13) ion mass signals were background corrected. From the relative fragment signal intensity ratios (m/z =27 : m/z =26) of ethylene and ethane (see Fig. S12b or Table S1) the corresponding theoretically expected ion mass signals at m/z=26 "Tho" was derived for pure C_2H_4 contribution at m/z =27 (light blue in Figure 4a,b Figure S13) and pure C_2H_6 contribution at m/z =27 (light grey in Figure 4a,b Figure S13), respectively. The singly ^{13}C labelled ethylene fragment, $^{13}CCH+$ at m/z=26 was neglected due to its negligible contribution of about 0.1%. The resulting theoretically expected m/z=26 "Tho" ion mass signal for any ethylene to ethane product ratio falls into the grey hashed areas of in Figure 4a,b and between the blue and grey traces in Figure S13.

$$I_{\text{m/z 26 (ethylene)}} = I_{\text{m/z 27 (ethylene)}} \text{ x } 0.84 \quad \text{(S13)}$$
$$I_{\text{m/z 26 (ethane)}} = I_{\text{m/z 27 (ethane)}} \text{ x } 0.70 \quad \text{(S14)}$$

The fact that the experimental m/z = 26 ion mass signal (red in Figure 4 and Figure S13) significantly exceeds the grey hashed area (or area between blue and grey) suggests a significant additional contribution at m/z = 26, which we associate with the molecular ion mass of acetylene, C_2H_2. Note that consideration of any additional contributions of further (minor) products at m/z =27, perhaps acetaldehyde, would further increase the acetylene contribution at m/z =26, as it would lower the combined contributions of ethane and ethylene to m/z =26. We consider our extracted acetaldehyde intensity as a conservative lower limit.

$$I_{\text{m/z 26 (Experimental)}} = I_{\text{m/z 26 (ethylene)}} + I_{\text{m/z 26 (ethane)}} + I_{\text{m/z 26 (acetylene)}} \quad \text{(S15)}$$

Assuming m/z = 27 stems entirely from ethylene, the corresponding acetylene, C_2H_2, -related mass signal at m/z (C2H2+) = 26 is extracted and, as 100% peak, normalized by its relative sensitivity factor (RSF) of 1.28 (see Table S1). This results in the normalized molecular ion mass signal "i_{Norm}" (green MSCV in Figure 4a,b and Figure S13). Similarly, a normalized ion mass signal "i_{Norm}" for ethylene, evaluated at its 100% molecular ion mass m/z (C2H4+) = 28 is derived from the ethylene contribution at m/z (C2H2+) = 26 and m/z (C2H3+) = 27 using a RSF of 1.02 (orange line in Fig. 3a,b and Figure

S13). The ratio of integral i_{Norm} ion mass charges of acetylene and ethylene in Figure 4a,b range at 7:100 for both catalysts (7 mol %). This ratio represents the ratio of partial pressures and molar product amounts in the ion source of the mass spectrometer. Assuming steady state product concentrations in the DEMS capillary sampling, this value represent the chemical selectivity of acetylene at the point of capillary sampling near the catalyst interface.

1.1.8 Relative Sensitivity Factors, RSF, and normalized ion mass currents i_{Norm}

The mass spectrometric relative sensitivity factor, RSF_j, of a compound j of a mass spectrometer represents a relative conversion factor between the partial pressure or molar flow of j at the ion source of a mass spectrometer and the measured ion mass current at the detector.[6-8] RSF_j values are related to absolute sensitivity factors K_j through the sensitivity of a reference compound, typically N_2 according $RSF_j = K_j/K_{N2}$.[3] RSF_j values include the ionization probability of molecule j at the ion source, R_S (which correlates with the number of electrons of the molecule), the fragmentation factor, R_F, the Quadrupole transmission factor, R_Q, and the detection efficiency, R_D according to[6]

$$RSF_j = R_S R_F R_Q R_D \, . \qquad \text{(S16)}$$

The source sensitivity, R_S, the fragmentation factor, R_F can be estimated from generic data. , R_S depends on the molecular mass and the electron impact energy. R_F simply follows from fragmentation patterns obtained at the electron impact energy. By convention, R_F values refer to the fragment with 100% intensity, which is why RSF values should exclusively be applied to that ion mass fragment of compound j. The transmission factor, R_Q, and the detection efficiency, R_D, depend on the instrument design. Values are available for common instrument designs. Over the years, Mass spectrometer manufacturer have measured or estimated RSF data and have listed the RSF data for their mass spectrometers in the technical literature (see Table S1).[3, 6-9]

Normalized ion mass currents, i_{Norm}, of compound j are obtained from measured or deconvoluted ion mass currents of the 100% intensity fragment, $i_{MS,j}^{100\,\%}$, by dividing with the RSF factor of compound j according to

$$i_{Norm} = i_{MS,j}^{100\,\%}/RSF_j \qquad \text{(S17)}$$

The integral of i_{Norm} corresponds to a molar amount of j. Ratios of areas under i_{Norm} -based Mass Spectrometric cyclic voltammograms (MSCVs) (integrated over time) represent relative molar (production) rates and chemical selectivities.

1.2 DFT Calculational Details

Density functional theory (DFT) calculations were performed using the Vienna ab initio simulation package (VASP).[10, 11] The interactions between electron and ion were described using the projector augmented wave (PAW) method[12] and the generalized gradient approximation was adopted by the revised Perdew−Burke−Ernzerhof (RPBE) functional.[13] The cutoff energy of plane-wave basis sets was set to be 400 eV, and convergence criteria for structural optimization were 10^{-5} eV (energy) and 0.01 eV/Å (force), respectively. The climbing image nudged elastic band method (CINEB)[14, 15] was used to calculate the kinetic barrier of acetylene coupling, where the convergence criteria were set as 10^{-4} eV and 0.02 eV/Å for energy and force. Four-layer slab models were built as 4*4 cell of Cu(111) surface with a total number of 64 Cu atoms, where the bottom two layers were fixed. The Brillouin zones were sampled by γ-centered meshes of 3 × 3 × 1 grids and vacuum layers of around 16 Å were added to all slab models. The free energy modification was evaluated with the VASPKIT package.[16]

2. Experimental DEMS Results

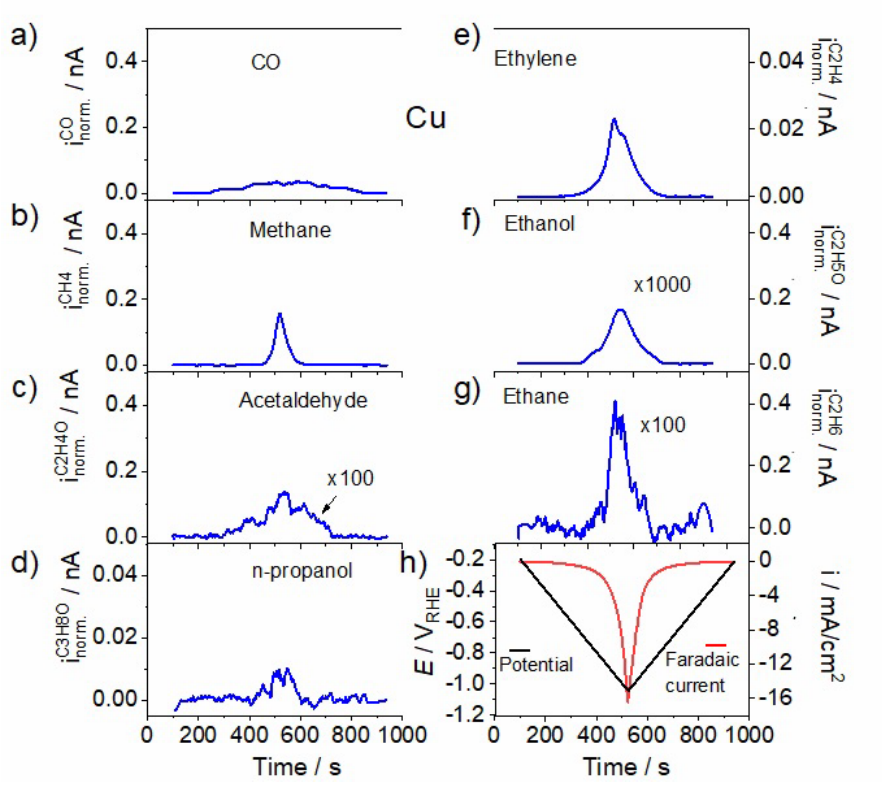

Supplementary Figure S7: Mass spectrometric cyclic voltammograms (MSCV) during CO2RR and experimental onset potentials on the metallic Cu catalyst ("Cu"): The Figures a-g show the time-resolved ion mass currents vs time during an applied voltammetric electrode potential cycle, recorded during CO_2 electroreduction on the polycrystalline metallic copper (Cu) catalyst in CO_2-saturated 0.1 M $KHCO_3$. a-g) The reaction products of the CO2RR were assigned using their characteristic mass-to-charge ratios signals (m/z). All MSCV ion mass currents of products were plotted in the time domain (blue curves). a) Carbon monoxide (CO) at m/z = 28 at main ion fragment CO^+ (100%), b) Methane (CH_4) at m/z = 15 with fragment CH_3^+ (85%), c) Acetaldehyde at m/z = 29 with fragment CHO^+ (100%), d) 1-propanol (C_3H_8O) at m/z = 60 with fragment $C_3H_8O^+$ (6,5%), e) Ethylene (C_2H_4) at m/z = 26 with fragment $C_2H_2^+$ (55%), f) Ethanol (EtOH) at m/z = 31 with fragment $-CH_2OH^+$ (100%) and g) Ethane (C_2H_6) at m/z = 30 with fragment $C_2H_6^+$ (26%). h) The triangular potential scan in the time domain (black, -0.2 V_{RHE} to -1.1 V_{RHE} at scan rate of 2 mV/s, CO_2 partial pressure was 100 kPa (P_{CO2} = 100 kPa)) and the corresponding faradaic current (red) are plotted together.

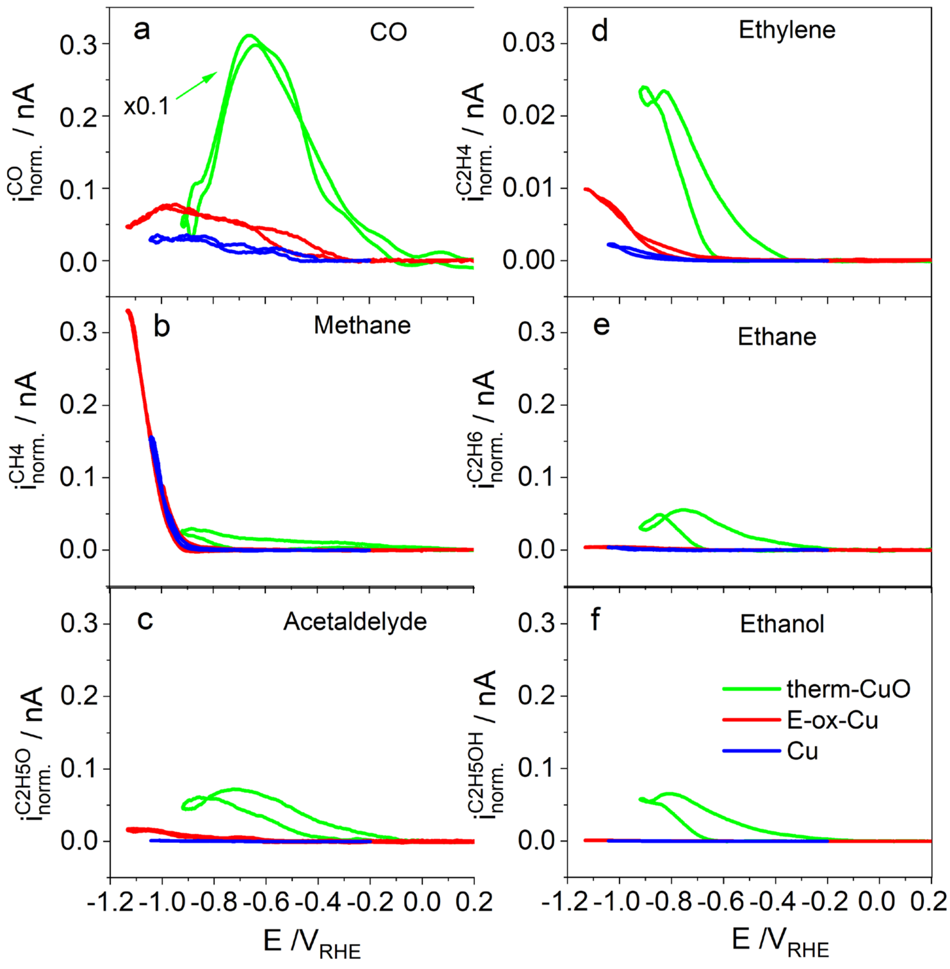

Supplementary Figure S8: Comparative Mass spectrometric cyclic voltammograms (MSCV) and product onset potentials plotted in the potential domain, the Figure directly compares the thermally oxidized Cu catalyst (**therm-CuO**) in green, electrochemically surface oxidized Cu catalyst (**E-ox-Cu**) in red, and the oxide-free polycrystalline metallic Cu catalyst (**Cu**) in blue during bulk CO_2 electrolysis. The CO2RR products shown were identified and plotted selected one individual deconvoluted mass-to-charge ratio (m/z) peak. a) **Carbon monoxide** (CO) at m/z= 28 at main ion fragment CO^+ (100%), b) **Methane** (CH_4) at m/z =15 with fragment CH_3^+ (85%), c) **Acetaldehyde** at m/z= 29 with fragment CHO^+ (100%), d) **Ethylene** (C_2H_4) at m/z = 26 with fragment $C_2H_2^+$ (55%), e) **Ethane** (C_2H_6) at m/z = 30 with fragment $C_2H_6^+$ (26%), f) **Ethanol** (EtOH) at m/z =31 with fragment -CH_2OH^+ (100%) The protocol multiple ion detection was used for detection of all mass spectrum signals. The cyclic potential scan started at -0.2 V_{RHE} at scan rate of 2 mV/s and went to -0.9 V_{RHE} for **therm-CuO**, to -1.1 V_{RHE} for **E-ox-Cu**, and to -1.0 V_{RHE} for the metallic **Cu** catalysts; electrolyte was CO_2 saturated 0.1 M $KHCO_3$ at a CO_2 partial pressure of 100 kPa (P_{CO2} = 100 kPa).

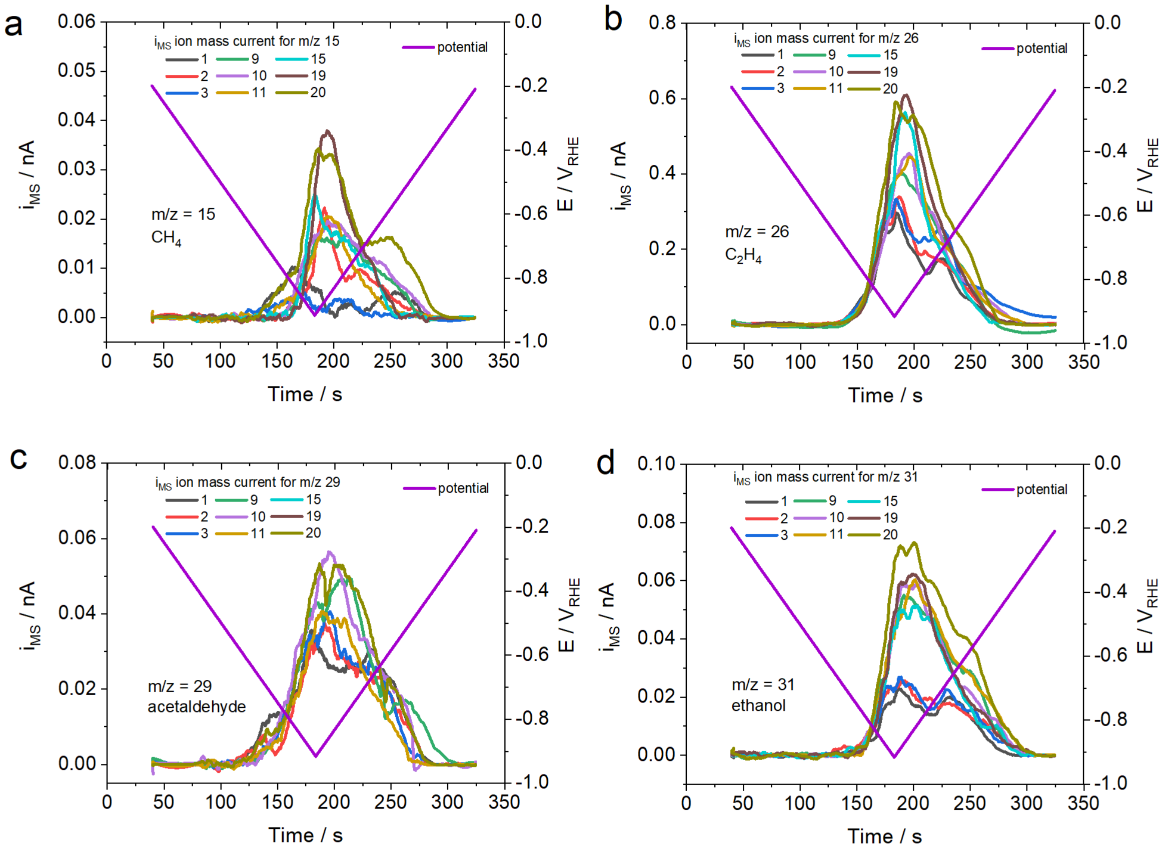

Supplementary Figure S9: **MSCVs and Onset potential stability during prolonged cyclic potential scans on the "therm-CuO" catalyst**: Experimental MSCVs during selected consecutive potential cycles (1-20) for four major volatile CO2RR reaction products on **therm-CuO** catalysts during CO2RR. a) **Methane** at m/z= 15, b) **Ethylene** at m/z =26, c) **Acetaldehyde** at m/z=29 and d) **Ethanol** at m/z =31. CO2RR was performed in CO_2 saturated 0.1 M bicarbonate electrolyte (P_{CO2} = 100 kPa). The protocol multiple ion detection was used for detection of all mass spectrum signals. The applied cyclic electrode potentials are shown in purple (right y axis). The potential was cycled between -0.2 V_{RHE} and -0.9 V_{RHE} at 5 mV/s.

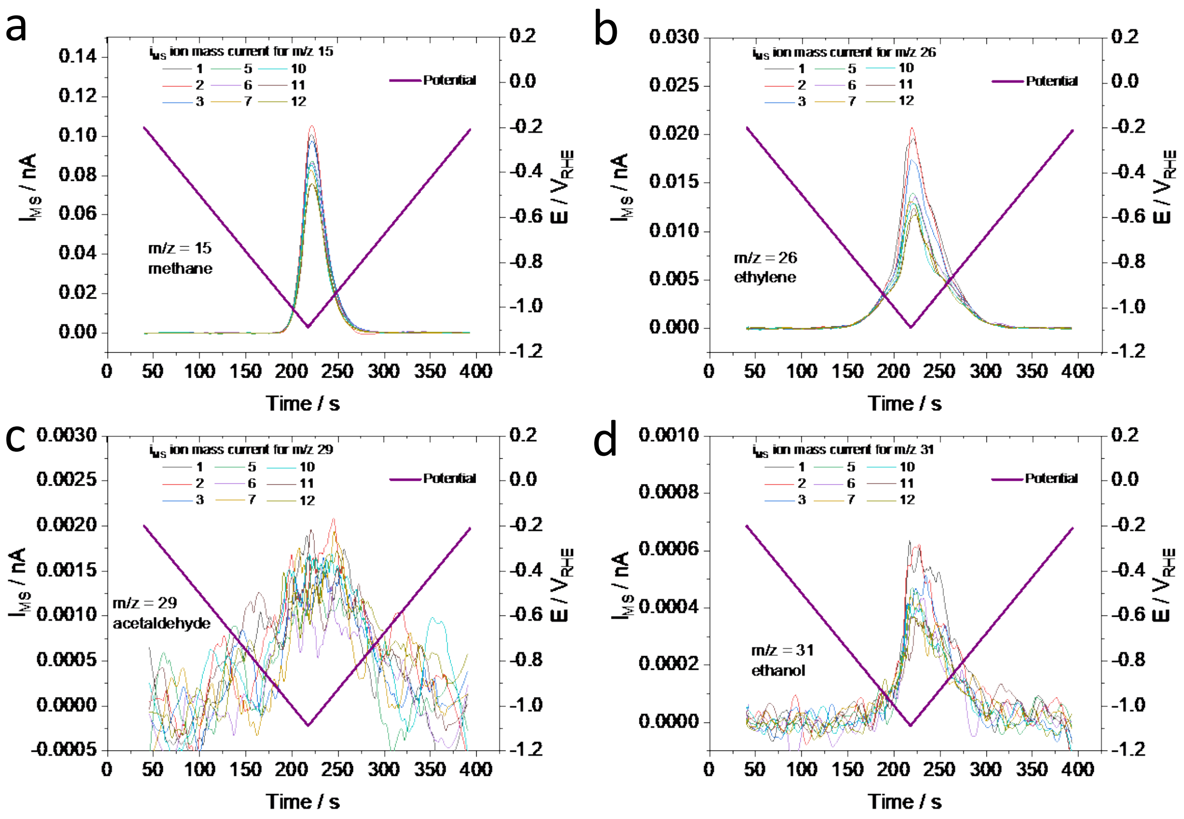

Supplementary Figure S10: **MSCVs and Onset potential stability during prolonged cyclic potential scans on the "E-ox-Cu" catalyst**: Experimental MSCVs during selected consecutive potential cycles (1-20) for four main volatile CO2RR reaction products on **therm-CuO** catalysts during CO2RR. a) **Methane** at m/z= 15, b) **Ethylene** at m/z =26, c) **Acetaldehyde** at m/z=29 and d) **Ethanol** at m/z =31. CO2RR was performed in CO_2 saturated 0.1 M bicarbonate electrolyte (P_{CO2} = 100 kPa). The protocol multiple ion detection was used for detection of all mass spectrum signals. The applied cyclic electrode potentials are shown in purple (right y axis). The potential was cycled between -0.2 V_{RHE} and -1.1 V_{RHE} at 5 mV/s.

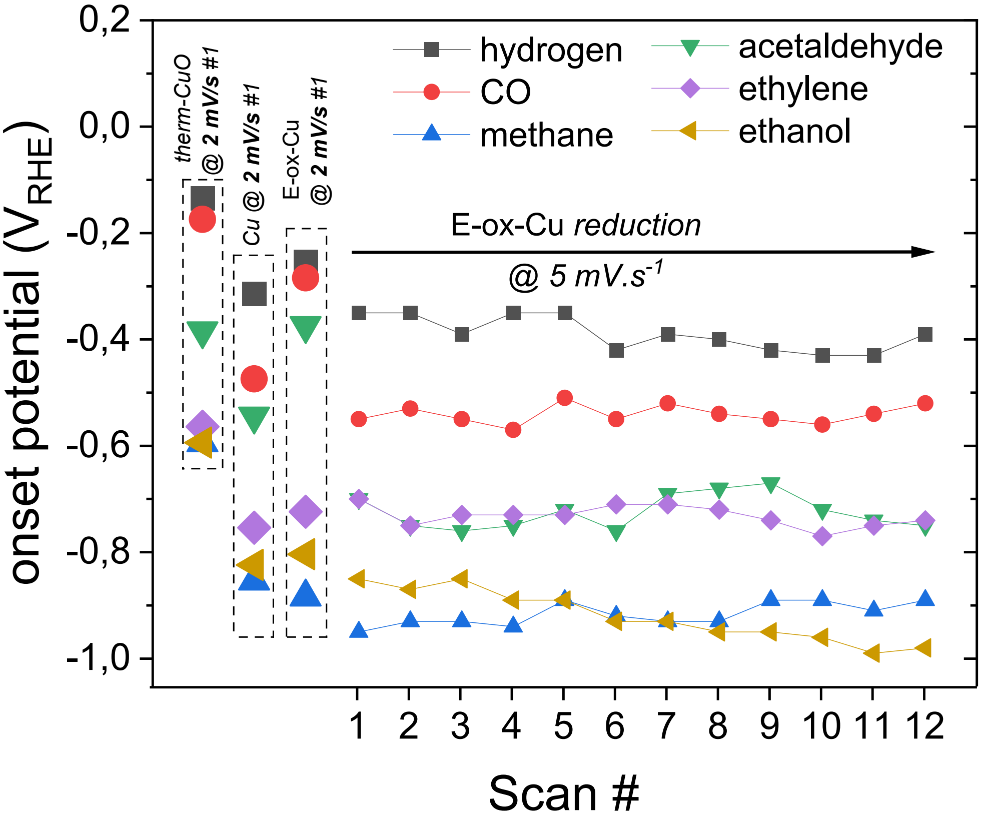

Supplementary Figure S11: **Comparative initial onset potentials and the evolution/stability of product onset potentials. Dashed Boxes**: Experimental onset potentials (in V_{RHE}) taken from the cathodic portion of the initial MSCVs (here scan #0) recorded using a scan rate of 2 mV/s for the thermally oxidized Cu-oxide ("therm-CuO"), the metallic oxide-free polycrystalline copper ("Cu") catalysts, and the anodically oxidized polycrystalline copper ("E-ox-Cu"), see the labels on top of dashed boxes. Color symbols refer to distinct CO2RR reaction products in legend. **Right portion of plot**: Time variation of the product onset potentials for the "E-ox-Cu" catalyst. The catalyst was subjected to consecutive 12 voltammetric cycles between -0.2 V_{RHE} to -1.1 V_{RHE} at a scan rate of 5 mV/s. The onset potential was defined as the potential where ion current signals reached 1% of the peak intensity for each product during a cathodic sweep. Electrolyte conditions: in 0.1 M KHCO3 saturated by 1 bar flowing CO_2.

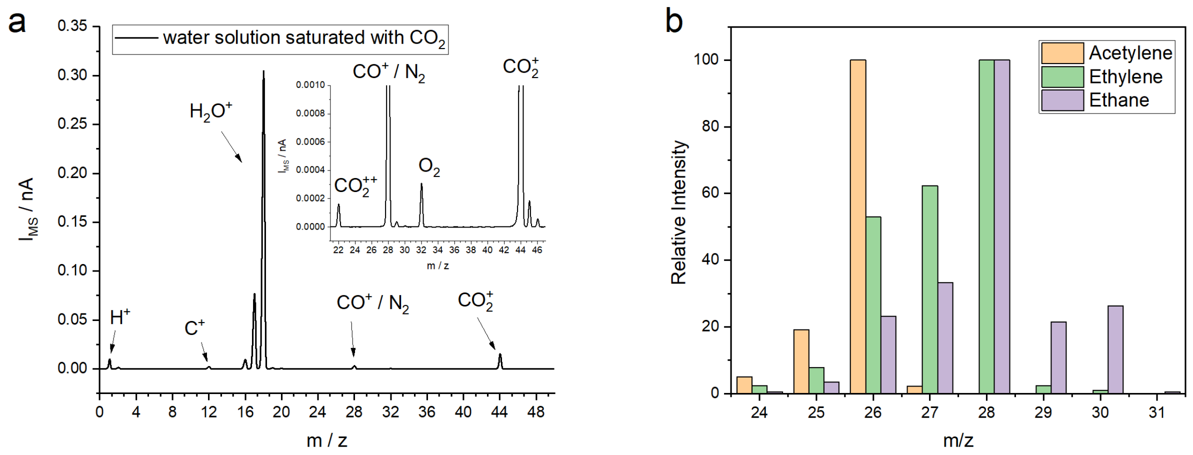

Supplementary Figure S12: Relative intensity fragmentation in mass spectrum analysis. a) a typical background mass spectrum in CO_2 saturated aqueous bicarbonate solution during the electrochemical CO2RR, measured directly at the DEMS capillary flow cell. Inset: blow up of the ion mass range m/z= 22 to 44, showing the m/z = 26-27, m/z = 29-31, and m/z(CO+/N2+) = 28 and its background peaks from the electrolyte relevant for deconvolution of major C_2 products during CO2RR; b) the relative peak intensities of the mass spectra of pure acetylene, ethylene and ethane taken from https://webbook.nist.gov/. The data apply to electron impact ionization at 70 eV. The relative contributions to the various m/z signals are used to deconvolute the CVMS of individual products.

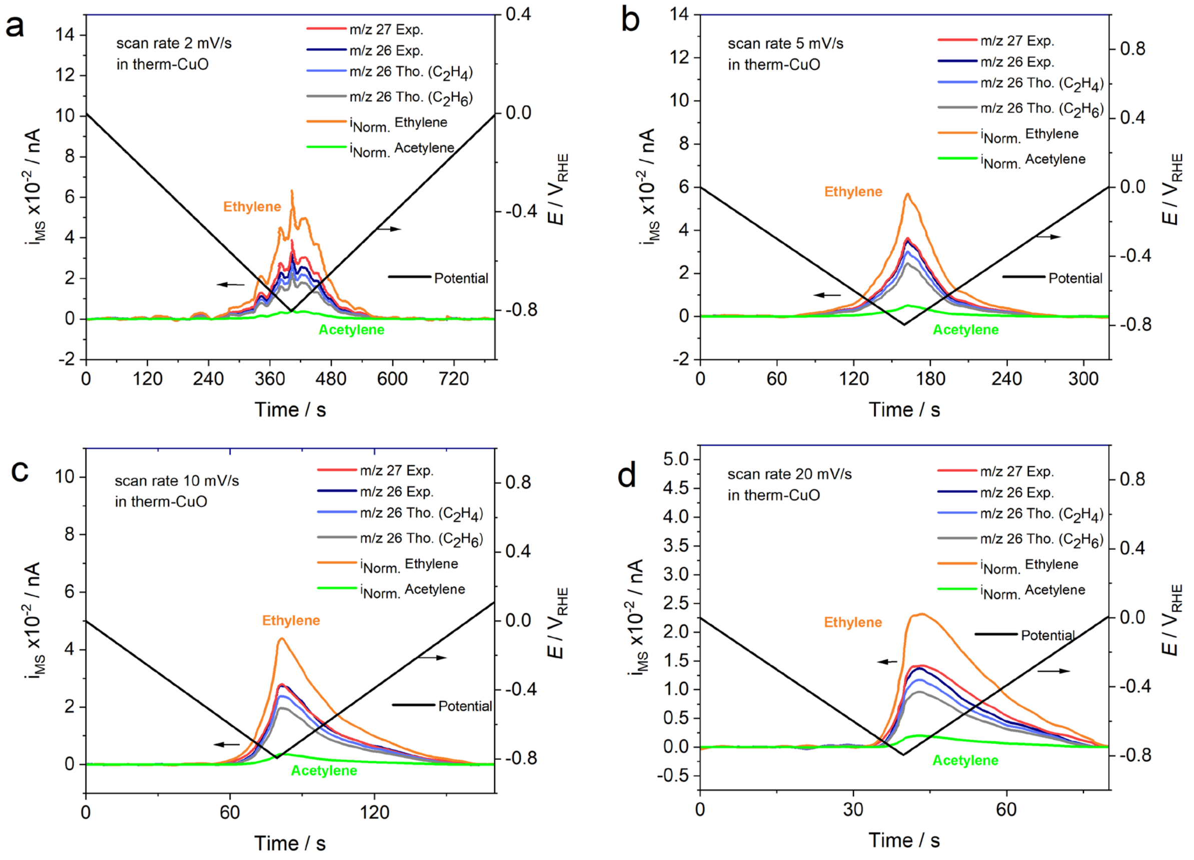

Supplementary Figure S13: Scan rate dependence study of the CO_2 electroreduction to Acetylene, C_2H_2, during cyclic voltammetry on the thermally oxidized Cu-oxide disk ("therm-CuO" catalyst). Shown are Mass spectrometric cyclic voltammograms (MSCVs) of the recorded ion currents used for deconvolution and quantification of acetylene formation during CO2RR. a) 2 mV/s, b) 5 mV/s, c) 10 mV/s and d) 20 mV/s. The characteristic experimental (Exp.) mass signals, from which the acetylene mass signal is derived, are m/z = 26 (dark blue "m/z 26 Exp.") and m/z = 27 (red line "m/z 27 Exp."). The theoretically expected MSCVs at m/z = 26 assuming pure ethylene and ethane contributions at m/z = 27 are shown as the light blue line ("m/z 26 Theo. C_2H_4") and grey line ("m/z 26 Theo. C_2H_6"). The relative sensitivity factor-normalized (i_{Norm}) ion current of the 100 % fragment peak (see Table S1) of Acetylene (green line) and Ethylene (orange) are given. Deconvolution of acetylene at m/z 26, $C_2H_2^+$ (intensity 100%) , from ethylene fragments $C_2H_2^+$ (relative intensity of 55%) is achieved using its fragment intensity ratio relative to the ethylene m/z = 27 $C_2H_3^+$ fragments (relative intensity of 62%). The voltammetric potential cycle was applied starting at +0.54 V_{RHE} to -0.9 V_{RHE} (black triangular lines, right y axis); Reduced partial pressures of dissolved CO_2 gas in solution (P_{CO2} = 23 kPa) minimizes nearby m/z 28 CO^+ intensity.

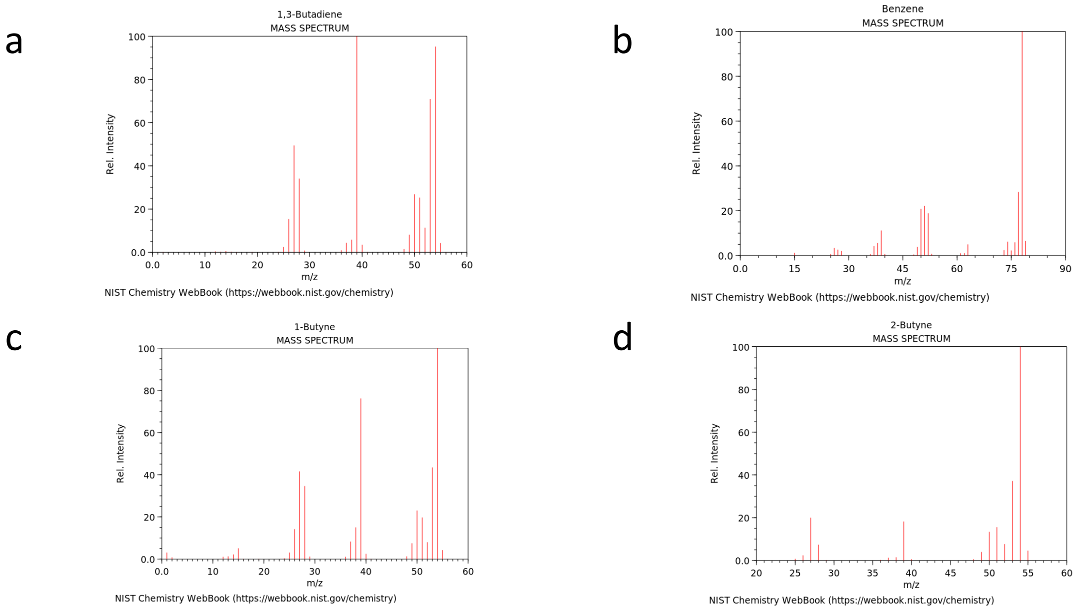

Supplementary Figure S14: Tabulated Mass Spectra of 1,3, Butadiene, benzene, 1-Butyne and 2-Butyne for assignment of experimentally observed C_4 product: Tabulated Fragmentations of a) 1,3 Butadiene b) benzene, c) 1-Butyne and c) 2-Butyne.

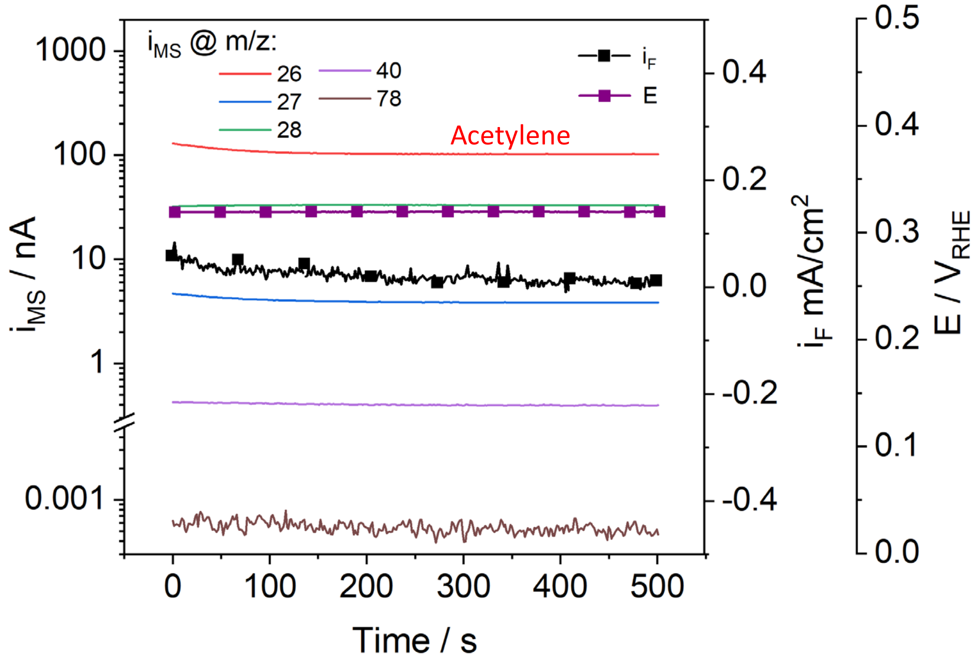

Supplementary Figure S15: Verification of the effect of applied interfacial electrode potential on the electro-trimerization of acetylene to benzene on Cu surfaces: Open Circuit Potential (OCP) measurement on polycrystalline copper (Cu) with simultaneous ion mass current signal recorded on m/z= 26 (Acetylene), m/z= 27 (ethylene), m/z= 28 (CO/N2), m/z= 40 (Ar), and m/z= 78 (benzene). Continuous diluted acetylene feeding in 0.1 KOH electrolyte solution ($P_{acetylene}$ = 23 kPa). The dilution minimized acetylene polymerization reactions on surface.

3. Physical and Chemical Characterizations

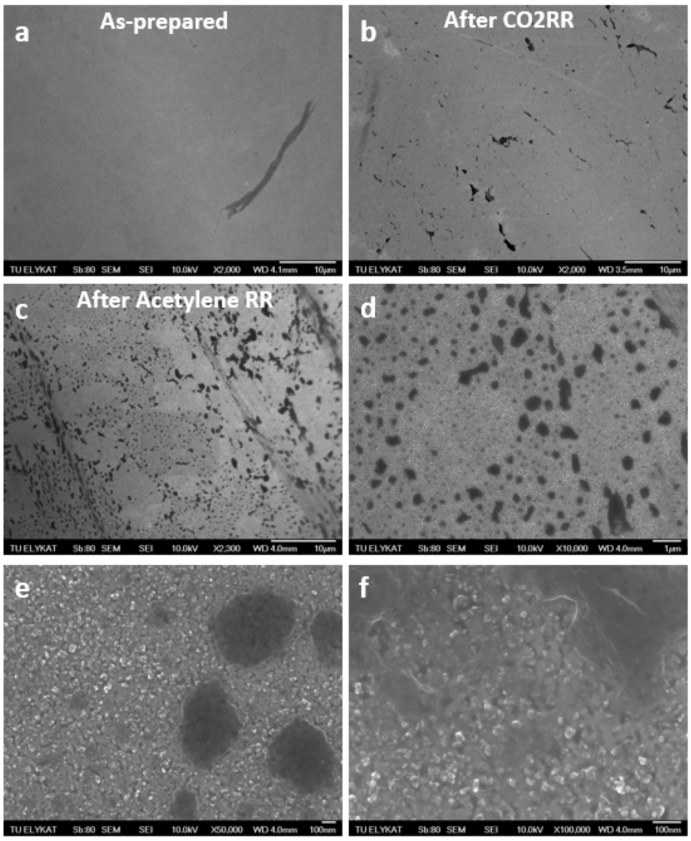

Supplementary Figure S16: Catalyst surface morphology after CO2RR and Acetylene di-trimerization: SEM micrographs of a Cu electrode catalyst surface at and after various reaction conditions. a) the as-prepared polished polycrystalline Cu surface before electroreduction, b) catalyst surface after CO_2 reduction reaction (CO2RR) at p_{CO2}= 100 kPa (conditions as in Fig. 2), c-f) various locations and magnifications of the Cu electrode surface after acetylene reduction reaction (Fig. 5c,d), p_{C2H2}=23 kPa

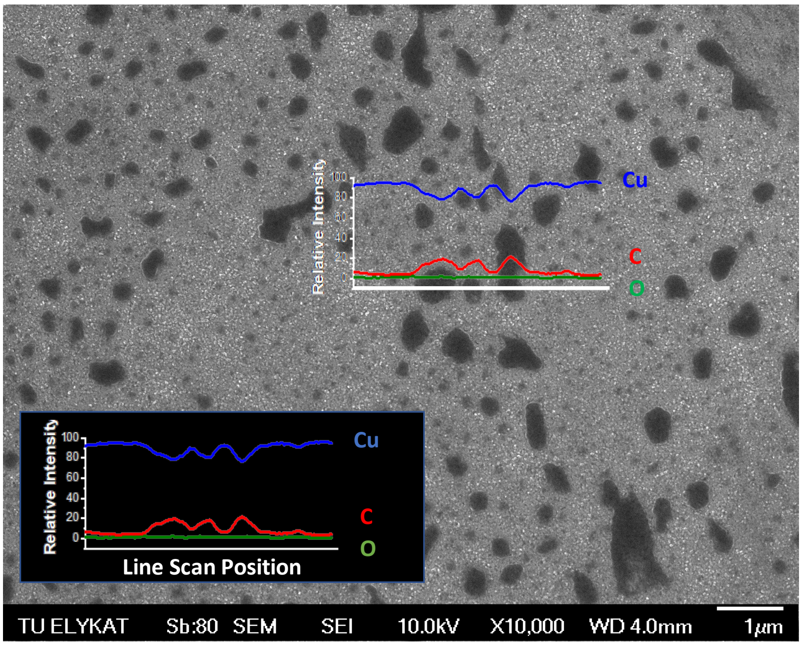

Supplementary Figure S17: **Surface Composition and morphology after acetylene di-trimerization**: SEM and line scan EDX analysis (horizontal white line in center of image) of Cu, C, and O fluorescence on the surface of a Cu electrode after Acetylene electroreduction reaction (conditions of Fig. 5c,d). insets: show the relative intensity of the elemental EDX signals of Cu, C, and O along the scanned line. For better readability and visibility, the EDX line scan is reproduced on a black background in the lower left corner of the image.

.

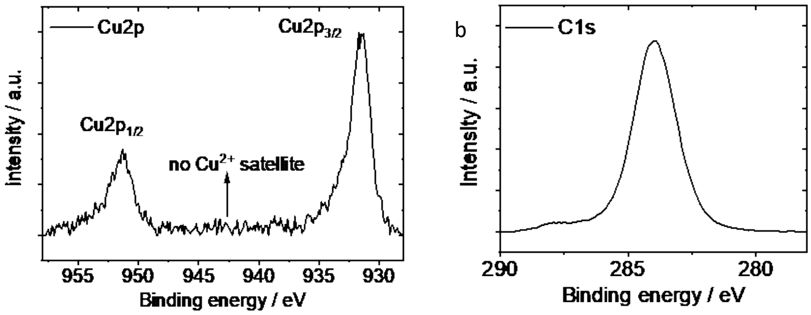

Supplementary Figure S18: Chemical State of the Cu surface after acetylene cyclo-trimerization: High resolution X-ray Photoemission Spectra in the Cu2p (a) and C 1s (b) range of a Cu catalyst surface after acetylene reduction reaction (conditions as in Fig. 5c,d).

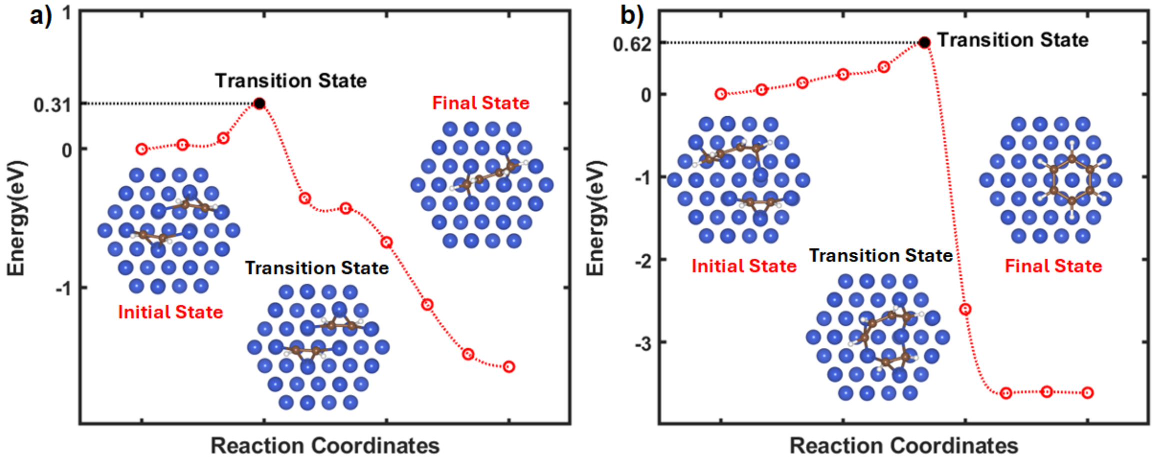

Supplementary Figure S19: Reaction barriers and atomic configurations of key reaction species in NEB calculations: (a) barriers along the reaction "$*C_2H_2 + *C_2H_2 \rightarrow *C_4H_4$" and (b) barriers along the reaction "$*C_2H_2 + *C_4H_4 \rightarrow *C_6H_6$". Color map: blue—Cu, brown—C, white—H.

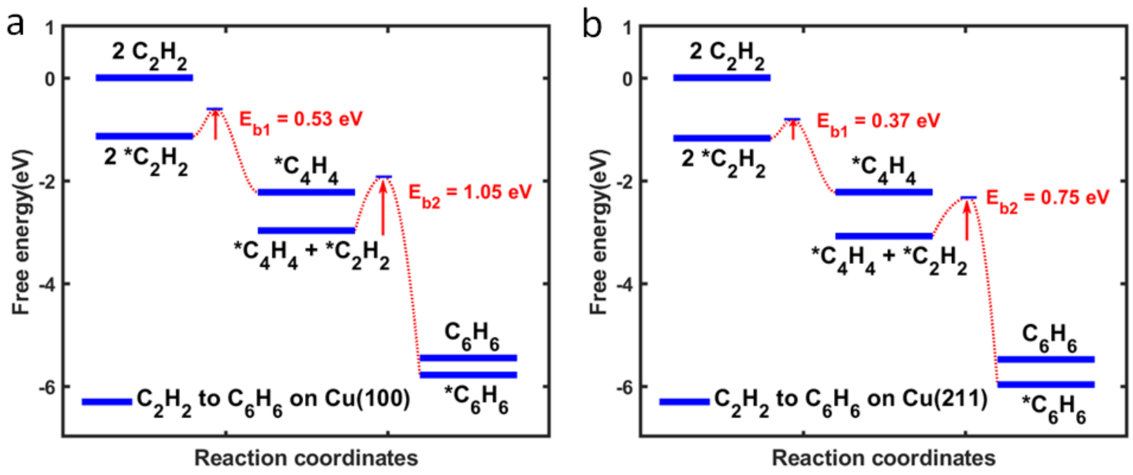

Supplementary Figure S20: **Additional Free energy diagrams for benzene formation on surfaces other than (111):** (a) Free energy diagram of acetylene reaction to benzene (3 C_2H_2 → C_6H_6) with barriers on Cu(100). $2C_2H_2$ to C_4H_4 has a barrier of 0.53 eV and C_2H_2+C_4H_4 to C_6H_6 (benzene) has a barrier of 1.05 eV, both of which are much higher than those on Cu(111). (b) Free energy diagram of acetylene reaction to benzene (3 C_2H_2 → C_6H_6) with barriers on Cu(211). The barriers of both coupling steps are comparable to those on Cu(111) yet C_6H_6 desorption is more difficult than Cu(111).

Supplementary Table S2: Experimentally determined onset potentials (in $\mathbf{V_{RHE}}$) of various CO_2 reduction products on polycrystalline oxidic "therm-CuO" catalysts, surface oxidic "**E-ox-Cu**" catalysts and poly crystalline metallic "**Cu**" catalysts during cathodic potential scans at **2mV/s** in CO_2 -saturated 0.1 M $KHCO_3$ electrolytes (P_{CO2}= 100 kPa) at ambient temperature.

Product/Catalyst	Therm-CuO	E-ox-Cu	Cu
**Hydrogen H_2 (m/z = 2)**	-0.13 ± 0.01	-0.25 ± 0.02	-0.31 ± 0.05
**Carbon monoxide CO (m/z = 28)**	-0.17 ± 0.06	-0.28 ± 0.02	-0.47 ± 0.08
**Methane CH_4 (m/z = 15)**	-0.59 ± 0.04	-0.85 ± 0.01	-0.83 ± 0.01
**Acetaldehyde CH_3CHO (m/z = 29)**	-0.38 ± 0.05	-0.37 ± 0.03	-0.75 ± 0.08
**Ethylene C_2H_4 (m/z= 26,27)**	-0.56 ± 0.03	-0.72 ± 0.02	-0.71 ± 0.01
**Ethanol CH_3CH_2OH(m/z = 31)**	-0.59 ± 0.02	-0.81 ± 0.01	-0.82 ± 0.04
**Ethane C_2H_6 (m/z = 26,27,28,30)**	-0.57 ± 0.03	-0.69 ± 0.05	-0.81 ± 0.04
**Acetylene C_2H_2 (m/z = 26)**	-0.55 ± 0.05	-0.67 ± 0.04	-0.70 ± 0.03
**Propylene CH_3CHCH_2 (m/z = 39,41,42)**	-0.60	--	--
**Propionaldehyde CH_3CH_2CHO (m/z = 58,60)**	-0.52	--	--
**n-Propanol $CH_3CH_2CH_2OH$ (m/z = 58,60)**	-0.70	--	--

Supplementary Table S3: Experimentally determined onset potentials (in V_{RHE}) of C_4 and C_6 reduction products (1,3 butadiene and benzene) on polycrystalline oxidic "therm-CuO" catalysts (Figure 5c) and surface oxidic "E-ox-Cu" catalysts (Figure 5d) during cathodic potential scans at 5 mV/s at ambient conditions.

Product/Catalyst	Therm-CuO	E-ox-CuO
Ethylene C_2H_4 (m/z= 27)	-0.14	-0.55
1,3 butadiene $CH_2CHCHCH_2$ (m/z = 54)	-0.19	-0.48
Benzene C_6H_6 (m/z = 78)	-0.18	-0.44

4. References

(1) Hoffmann, E. d.; Stroobant, V. *Mass Spectrometry - Principle and Applications*; Wiley, 2007.
(2) Gross, J. H.; Springer International Publishing, A. G. *Mass Spectrometry : A Textbook*; 2017.
(3) Jousten, K. *Handbuch of Vakuumtechnik*; Springer Vieweg, 2018.
(4) Wolter, O.; Heitbaum, J. Differential Electrochemical Mass Spectroscopy (DEMS) - a New Method for the Study of Electrode Processes. *Berichte der Bunsengesellschaft für physikalische Chemie* **1984**, *88* (1), 2-6. DOI: 10.1002/bbpc.19840880103.
(5) Baltruschat, H. Differential electrochemical mass spectrometry. *Journal of the American Society for Mass Spectrometry* **2004**, *15* (12), 1693-1706. DOI: http://dx.doi.org/10.1016/j.jasms.2004.09.011.
(6) Relative Sensitivity and RS Measurement of Gases - Application Note 282. Hiden Analytical. (accessed 15.1.2022).
(7) *Pfeiffer Vacuum Application Notes Mass Spectrometry - https://www.pfeiffer-vacuum.com/de/know-how/massenspektrometer-und-restgasanalyse/quadrupol-massenspektrometer-qms/ionenquellen/* 2022. (accessed.
(8) *Fragmentation Patterns and Relative Sensitivity Factors - https://www.hidenanalytical.com/tech-data/cracking-patterns/* 2022. (accessed 16.1.2022).
(9) *NIST Chemistry WebBook - https://webbook.nist.gov/ (accessed 16.1.2022)*. NIST, 2022. (accessed.
(10) Kresse, G.; Furthmüller, J. Efficient iterative schemes for ab initio total-energy calculations using a plane-wave basis set. *Physical Review B* **1996**, *54* (16), 11169-11186. DOI: 10.1103/PhysRevB.54.11169.
(11) Kresse, G.; Hafner, J. Ab initio molecular dynamics for liquid metals. *Physical Review B* **1993**, *47* (1), 558-561. DOI: 10.1103/PhysRevB.47.558.
(12) Blöchl, P. E. Projector augmented-wave method. *Physical Review B* **1994**, *50* (24), 17953-17979. DOI: 10.1103/PhysRevB.50.17953.
(13) Hammer, B.; Hansen, L. B.; Nørskov, J. K. Improved adsorption energetics within density-functional theory using revised Perdew-Burke-Ernzerhof functionals. *Physical Review B* **1999**, *59* (11), 7413-7421. DOI: 10.1103/PhysRevB.59.7413.
(14) Henkelman, G.; Uberuaga, B. P.; Jónsson, H. A climbing image nudged elastic band method for finding saddle points and minimum energy paths. *The Journal of Chemical Physics* **2000**, *113* (22), 9901-9904. DOI: 10.1063/1.1329672 (acccessed 2/12/2025).
(15) Henkelman, G.; Jónsson, H. Improved tangent estimate in the nudged elastic band method for finding minimum energy paths and saddle points. *The Journal of Chemical Physics* **2000**, *113* (22), 9978-9985. DOI: 10.1063/1.1323224 (acccessed 2/12/2025).
(16) Wang, V.; Xu, N.; Liu, J.-C.; Tang, G.; Geng, W.-T. VASPKIT: A user-friendly interface facilitating high-throughput computing and analysis using VASP code. *Computer Physics Communications* **2021**, *267*, 108033. DOI: https://doi.org/10.1016/j.cpc.2021.108033.
